# Heterogeneities by Age and Sex in Associations Between Electronic Device Use Frequency and Comorbid Mental Health Symptoms: The Roles of Self-Efficacy and Social Support

**DOI:** 10.3390/healthcare14193140

**Published:** 2026-09-22

**Authors:** Xiao-Dong Qin, Li Lu, Rong Li, Zi-Wei Li, Zhongliang Zhou, Qing Shen, Sha Lai, Shou Liu

**Affiliations:** 1 Institute of Health Management and Policy, School of Public Policy and Administration, Xi’an Jiaotong University, Xi’an 710049, China; qxd1113543138@stu.xjtu.edu.cn (X.-D.Q.); lirong20230619@163.com (R.L.); ziweili@stu.xjtu.edu.cn (Z.-W.L.); zzliang1981@xjtu.edu.cn (Z.Z.); laisha@xjtu.edu.cn (S.L.); 2 System Behavior and Management Laboratory, Philosophy and Social Sciences Laboratory of the Ministry of Education, Xi’an Jiaotong University, Xi’an 710049, China; 3 Clinical Research Center for Mental Disorders, Shanghai Pudong New Area Mental Health Center, Tongji University School of Medicine, Shanghai 200092, China; qingshen@tongji.edu.cn; 4 Institute for Advanced Study, Tongji University, Shanghai 200092, China; 5 Unit of Integrative Epidemiology, Institute of Environmental Medicine, Karolinska Institutet, Solnavägen 1, 171 77 Stockholm, Sweden; 6 Department of Public Health, Medical College, Qinghai University, Xining 810016, China

**Keywords:** electronic device use, anxiety symptoms, depressive symptoms, self-efficacy, social support

## Abstract

**Highlights:**

**What are the main findings?**

- High-frequency mobile phone use was inversely associated with mental health comorbidities, particularly in young and middle-aged males, whereas low-to-mid frequency computer use was positively associated with comorbidities across male age groups.
- Weekly mobile phone use was associated with comorbid mental health symptoms primarily through indirect pathways, with self-efficacy and social support showing significant age- and sex-specific mediating roles.

**What are the implications of the main findings?**

- The buffering mechanisms of psychosocial resources against digital-related mental health risks are age- and sex-dependent.
- Interventions should shift from uniform device-use restrictions to age- and sex-tailored digital health strategies, providing actionable insights for specific stakeholders to address occupational technostress and improve population digital literacy.

**Abstract:**

**Objectives**: This study aims to examine age and sex disparities in the relationships between the weekly frequency of electronic device use and mental health comorbidities (anxiety symptoms, depressive symptoms, and stress), and the roles of self-efficacy and social support. **Methods**: Data were derived from the 2021 Psychology and Behavior Investigation of Chinese Residents (PBICR), which adopted a cross-sectional research design using a self-reported questionnaire-based survey. Random forest models were performed to examine the role of electronic device use on mental health, multinomial logistic regressions were conducted to quantify the associations, and mediation analyses via structural equation modeling were conducted to explore the roles of self-efficacy and social support, and all analyses were conducted by stratifying age and sex. **Results**: Of the 11,031 participants, 23.58%, 18.88%, and 16.91% of the young, the middle-aged, and older people reported mental health comorbidities, respectively. The weekly frequency of mobile phone use was identified as the second most important predictor of mental health comorbidities in adolescents and the young, as well as middle-aged adults. High-frequency mobile phone use was inversely associated with comorbidities, especially in young and middle-aged males. Low- and mid-frequency computer use were positively associated with comorbidity, especially in all males. The weekly frequency of mobile phone use played no significant direct role, and the significant direct association of weekly frequency of computer use was identified in middle-aged and older males. The mediating roles of self-efficacy and social support presented age and sex disparities. **Conclusions**: Despite the inherent limitations of cross-sectional, self-reported weekly data, the present findings highlight that policymakers and clinicians should consider the dual-edged role of digital device use in mental health, leveraging its benefits through tailored digital interventions while mitigating risks across diverse demographic groups.

## 1. Introduction

The pervasive proliferation of electronic devices has a profound impact on behavioral paradigms and social interaction modalities. It was shown that the global penetration rate of social media use surpassed 64% of the global population (5.20 billion users) [1], presenting a critical public health problem regarding its potential detriment to mental well-being. Electronic devices serve not exclusively as communication tools but rather as multifunctional platforms facilitating information acquisition, educational activities, and leisure pursuits. In the context of the accelerated evolution of digital transformation, society demands widespread adoption of remote work modalities and virtual learning environments [2], consequently amplifying people’s dependence on digital technologies. The modern digital ecosystem includes a broad spectrum of intelligent devices, such as mobile phones, laptops, tablets, smartwatches, and gaming consoles. The present study narrows its analytical scope to mobile phones and computers since the two devices dominate the majority of daily screen time, digital engagement, and online social and operational interactions among Chinese populations [3].

The widespread application undoubtedly draws attention to the intricate associations between electronic device use and mental health outcomes across developmental continua. For example, the use of digital technologies was noted to be associated with reduced risk of cognitive impairment and reduced time-dependent rates of cognitive decline [4], problematic electronic device use presented significant correlations with psychopathological manifestations encompassing depressive symptoms [5], and mental health outcomes were associated with sedentary digital consumption such as binge-watching [6,7]. Longitudinal evidence from the Adolescent Brain Cognitive Development (ABCD) Study has indicated that increased digital engagement was prospectively related to internalizing and externalizing symptoms in adolescents [8]. Notably, distinct sex-based patterns in digital behavior, such as higher gaming frequency among males and more social media engagement among females, further differentially shape mental health trajectories [9]. In addition, this association manifested substantial age-dependent variability, i.e., middle-aged and older Chinese populations demonstrated attenuated risk of depression concomitant with moderate internet engagement [10], in contradistinction to heightened psychopathology observed in digitally over-engaged adolescent cohorts, presenting age discrepancies. In addition, males and females exhibited different behavioral patterns and motivations of Internet usage. For example, these disparities permeated university populations, wherein problematic mobile phone usage predicted subsequent mental health deterioration, with stronger associations observed in females than in males [11], suggesting complex age- and sex-graded effects. With the rising prevalence of mental health symptoms and their substantial global burden on individuals and healthcare systems, addressing the association between digital device use and mental health is of great practical and clinical significance. Mental health comorbidity has become one of the core focuses [12,13,14], and the rationale for conceptualizing anxiety, depressive symptoms, and perceived stress as a comorbid triad is theoretically grounded. First, exposure to stressful stimuli typically elicits a spectrum of negative affective responses encompassing anxiety and depressive symptomatology, rather than isolated psychological disturbances [15]. Second, following the diathesis–stress theory, adverse life stress constitutes a critical precipitant of depression [16]. Meanwhile, anxiety and depressive symptoms exhibit robust reciprocal covariation and symptomatic overlap across populations [17]. Therefore, the intrinsic interrelatedness and synergistic activation of the three domains explain the scientific rationale for integrating these affective symptoms into a comorbidity construct.

Self-efficacy, a central construct in social cognitive theory, refers to an individual’s belief in their capacity to achieve desired goals and successfully execute tasks [18]. Previous studies have linked self-efficacy to the modification of traditional health behaviors [19,20], yet accumulating evidence has extended its relevance to digital health contexts. Specifically, one recent study emphasized that digital self-efficacy served as a pivotal determinant of individuals’ capacity to self-regulate screen-use behaviors, manage technology-induced cognitive loads, and adaptively engage with intricate digital ecological environments [21]. Perceived social support is often defined as the perception of available support from others, including family, friends, and strangers, and has been reported as one of the most robust psychosocial predictors of physical and psychological health outcomes [22,23,24]. Both online and offline social support networks act as vital buffers against digital stressors, such as information overload, technostress, and virtual harassment [25,26]. Social cognitive theory [27] showed that there were bidirectional interactions between the environment, individual factors, and behavior, and the triadic system functions through feedback processes operating internally (e.g., self-evaluations) and externally (changes in environmental conditions). In this process, self-efficacy is a critical appraisal factor of self-evaluation and social support operates as a primary environmental resource, which raises the question of whether self-efficacy and social support could be possible mediating factors in the relationship between electronic device use and comorbid mental health symptoms.

However, several aspects remain underexplored in the existing literature. First, extensive studies predominantly focused on isolated mental health outcomes, and in-depth studies of complex comorbid patterns of psychological distress, especially their relationships with electronic device use, are underexplored. Second, developmental trajectories of electronic-device-use-associated mental health remain inadequately explored across the lifespan continuum. Third, the mediating roles of factors related to positive psychology, such as self-efficacy and social support, in age- and sex-specific vulnerabilities should be explored. Therefore, the present study aims to (1) explore how the frequency of electronic device use (specifically focusing on computers and mobile phones) is associated with mental health comorbidities (including anxiety symptoms, depressive symptoms, and perceived stress) across age–sex strata; and (2) further examine the roles of self-efficacy and perceived social support in the relationships across different age and sex subgroups. This study will extend the existing literature by delineating age- and sex-specific vulnerabilities and clarifying the potential buffering roles of self-efficacy and social support. These findings would provide population-specific evidence to partially inform the development of demographic-stratified digital health interventions for researchers, clinicians, and policymakers. Based on previous studies [10,11,28,29,30] and the social cognitive theory, we made hypotheses that (1) there would be age- and sex-specific associations between the weekly frequency of electronic device use and comorbid mental health symptoms (H1); and (2) self-efficacy and social support would mediate the relationships between the frequency of device use and mental health comorbidities (H2).

## 2. Materials and Methods

### 2.1. Participants

The data in this study were derived from the 2021 Psychology and Behavior Investigation of Chinese Residents (2021 PBICR), which adopted a cross-sectional research design using a self-reported questionnaire-based survey. The participants were recruited using a multistage sampling method and were invited face-to-face to complete the survey questionnaires via the online ‘Questionnaire Star’ platform (Changsha Ranxing Information Technology Co., Ltd., Changsha, China). The survey was conducted between 10 July 2021 and 15 September 2021, and covered participants from 120 cities across 32 provinces, autonomous regions, and municipalities (Table S1) in China [31]. The inclusion criteria for the current study were: (1) permanent residents of Chinese nationality; (2) aged 12 or above; (3) with Chinese reading skills. Individuals with confusion or mental disorders, who had participated in other studies with a similar topic, or who refused to participate were excluded [32]. All participants volunteered to participate in the study and provided informed consent. For those who qualified but had mobility limitations, non-intrusive assistance was provided by investigators to complete the questionnaire. The study proposal was approved by the Ethics Research Committee of the Health Culture Research Center of Shaanxi, China (No. JKWH-2021-01). The study was carried out in accordance with the principles of the Declaration of Helsinki. More details about the study design are provided in previous literature [31,32].

### 2.2. Measurement

#### 2.2.1. Social Demographic Characteristics

The social demographic characteristics (i.e., covariates) included in this study encompassed demographic, socioeconomic, and lifestyle factors. Specifically, these included age group (adolescents and young people [12–25 years], middle-aged people [26–59 years], and older people [60 years and older]), sex (female/male), nationality (the Han/others), religious belief (no/yes), educational level (primary school and below, middle school, college/university degree or above), residential areas (urban/rural), the number of residential properties owned by family (i.e., quantity of owner-occupied housing/apartment units owned by family; 0, 1, ≥2), household indebtedness (no/yes), monthly household income per capita (≤3000 Chinese Yuan [CNY], 3001–6000, 6001–8999, ≥ 9000), and number of siblings (no, one or more). Lifestyle factors included smoking status (non-smokers or ex-smokers/current smokers), alcohol consumption in the past year (no/yes), and body mass index (BMI). Physical activity was evaluated by the exercise component of the Chronic Disease Self-Management Study Measures (CDSMS) [33,34], which includes six activities; for each activity, participants were asked, “*During the past week, how long did you spend doing the activity?*”, with responses ranging from 0 [never] to 4 [more than 3 h/week], yielding a total score of 0 to 24. In the current study, we categorized the scores into three levels: no (0), moderate (1–10), and high (11–24). Eating behaviors were measured using the 7-item short-form Sakata Eating Behavior Scale [35]; each item was scored from 1 (strongly disagree) to 4 (strongly agree) with a total score of 7 to 28, and the higher the score, the unhealthier the eating behavior. In the current study, we categorized the scores into three levels by the 25th and 75th percentiles [36]: healthy (7–14), general (15–20), and unhealthy (21–28).

#### 2.2.2. Exposures

In this study, the two exposure variables were the weekly frequencies of electronic device use (never/low-frequency/mid-frequency/high-frequency), with weekly use of a computer (including a laptop) and a mobile phone analyzed separately. The weekly frequencies of computer and mobile phone use were assessed by asking two separate questions: “*How often did you generally use your computer (including a laptop)?*” and “*How often did you generally use your mobile phone?*”. The responses were never, occasionally (≤1 day/week), sometimes (2–3 days/week), often (4–5 days/week), and almost every day (6–7 days/week), which were categorized as never, low-frequency (1–3 days/week), mid-frequency (4–5 days/week), and high-frequency (6–7 days/week) for further analyses. It is important to acknowledge that this categorization primarily captures the weekly presence of device use rather than the actual volume, intensity, duration, or objectives of screen exposure. Nevertheless, from a behavioral perspective, weekly frequency serves as a valuable proxy for the habitual integration and regularity of digital technology access in daily life, representing a distinct behavioral dimension from total screen time by capturing how consistently devices are woven into daily routines [37].

#### 2.2.3. Outcomes

**Anxiety symptoms** experienced by the participants over the previous two weeks were assessed using the validated Chinese version of the Generalized Anxiety Disorder 7-item Scale (GAD-7; Cronbach’s α: 0.89) [38,39]. Each item ranges from 0 (never) to 3 (almost every day) with a total score of 0 to 21, and higher scores indicate greater severity. In the current study, we categorized individuals with a score of 10 or more into the group presenting anxiety symptoms, otherwise into the group with an absence of anxiety symptoms (total score < 10) [39].

**Depressive symptoms** experienced by the participants during the previous two weeks were evaluated by employing the validated Chinese version of the Patient Health Questionnaire 9-item Scale (PHQ-9; Cronbach’s α: 0.86) [40,41]. Each item was scored ranging from 0 (never) to 3 (nearly every day), with a total score ranging from 0 to 27, and the higher the score, the more severe the symptoms. Participants with a total score of 10 or more were classified as having depressive symptoms [42,43].

The **perceived stress** level of recruited individuals was quantified by using the 3-item Perceived Stress Scale (PSS-3; Cronbach’s α: 0.887) developed by the Chinese Family Newspaper Research Center [44]. The questionnaire consists of three items, which inquire about the respondent’s self-perceived ability to handle stress, the level of stress experienced in the past two weeks (including both family and work life), and the level of stress experienced in the past year (including both family and work life). Each item was scored on a six-point Likert scale: item 1 ranged from “*I am able to overcome stress*” (1 point) to “*I am constantly troubled by stress*” (6 points), while items 2 and 3 ranged from “No stress” (1 point) to “Extreme stress” (6 points). In the current study, we summed the scores of the three items, yielding a total score ranging from 3 to 18, where higher scores represented higher perceived stress levels. The participants were then dichotomized into two groups by the 25th percentile [36,45]: “stressed” (total score ≥ 8) and “unstressed” (total score < 8). Given that PSS-3 measures general psychological stress, a sensitive quartile-based threshold approach was employed in the present study. Participants with scores in the bottom 25% were categorized as “unstressed”, establishing a stringent baseline group characterized by minimal stress burden [36,45]. The remaining 75% of the distribution captured a broad spectrum of stress, which frequently coincides with or precedes the onset of anxiety and depressive symptoms. Importantly, this threshold was used exclusively as a study-specific categorization to distinguish relative stress burdens within our sample, and was not intended to represent an established clinical cutoff.

We then constructed the variable, i.e., comorbid mental health symptoms (no/one/two or more), based on the data of the aforementioned three mental health outcomes. Specifically, we coded the variable as 0 for the absence of any mental health symptoms, 1 for the presence of one mental health symptom, and 2 for cases indicating two or more mental health symptoms.

#### 2.2.4. Mediating Variables

Perceived social support was assessed using the validated Chinese version of the 12-item self-reported Perceived Social Support Scale (PSSS; Cronbach’s α: 0.84) across three domains: family, friends, and significant others [46,47]. Responses are rated on a 7-point Likert scale, ranging from 1 (strongly disagree) to 7 (strongly agree) with a total score of 12 to 84, with higher scores indicating higher perceived social support levels. We categorized the scores into three levels, including low (12–36), moderate (37–60), and high (61–84) for characteristic descriptions, and all subsequent mediation analyses were performed using the original continuous scores to preserve statistical precision.

Self-efficacy was assessed using the 8-item New General Self-Efficacy Scale (NGSES; Cronbach’s α: 0.794), adapted from the General Self-Efficacy Scale (GSES) [48,49]. Each item is rated on a five-point Likert scale ranging from 1 (strongly disagree) to 5 (strongly agree), yielding a total score of 8 to 40, where higher scores indicate greater self-efficacy levels. The total score was categorized into three levels (low: 8–20; moderate: 21–30; high: 31–40) for descriptive statistics, with the continuous forms utilized in the mediation models.

### 2.3. Statistical Analysis

Descriptive statistics were used to summarize the study population, with continuous variables presented as mean ± standard deviation (SD) and categorical variables as number and frequency (percentage). Sociodemographic characteristics were compared between individuals across age and sex groups using analysis of variance (ANOVA) for continuous variables and the chi-square (χ^2^) test for categorical variables. The analytical methods of this study also integrated machine learning techniques, multinomial logistic regression models, and structural equation modeling (SEM) to identify key predictors of mental health comorbidities, quantify the associations, and explore the mediating pathways.

Random forest (RF) models were employed to evaluate the most significant individual and social contextual factors for and, especially, the weekly frequency of electronic device use’s contribution to comorbid mental health symptoms [50]. RF is a high-performance and reliable machine learning algorithm that was widely used [51,52,53]. Rooted in the “decision trees” methodology, RF partitions data through sequential binary splitting of input features to generate terminal predictive outcomes [54]. By constructing multiple independent decision trees in parallel and aggregating ensemble predictions, the algorithm substantially reduces overfitting risk and improves predictive stability [54,55]. “Feature importance” represents a core output of an RF model, which quantifies the relative predictive contribution of individual variables, with higher values indicating greater predictive influence [56]. In this framework, feature importance was computed based on the Gini importance, which estimates the total reduction of the criterion attributable to each input feature across tree splits [57]. The full dataset was randomly split into a 70% training subset and a 30% validation subset for model development and evaluation. Model predictive performance was assessed via confusion matrix-derived accuracy and the receiver operating characteristic (ROC) curve with area under the curve (AUC), which could also help to facilitate mechanistic interpretation of individual feature contributions to the model prediction.

We then employed multinomial regression models to quantify the relationships between the frequency of electronic device use and comorbid mental health symptoms, by adjusting for covariates and reporting the results as odds ratios (ORs) with their 95% confidence intervals (CIs). In the framework of SEM, the PROCESS macro program (Version 4.2, Andrew F. Hayes, University of Florida, Gainesville, FL, USA) in IBM SPSS Statistics (Version 27, IBM Corp., Armonk, NY, USA) [58] was used to test the mediating roles of self-efficacy and social support in the relationships between the frequency of electronic device use and mental health symptoms by using PROCESS Model 4 [59]. When testing the mediating roles, mental health symptoms were constructed as two separate variables: “comorbid vs. no” and “one mental health symptom vs. no” [60], and the continuous scores of self-efficacy and perceived social support were inputted as mediating variables.

Statistical significance was determined with a significant α threshold of 0.05 (two-tailed). The analyses were conducted via R (Version 4.4.1, R Core Team, Vienna, Austria) through RStudio (version 2025.09.1, Posit Software, PBC, Boston, MA, USA) for descriptive statistics, random forest models, and graphing, via Stata (version 17.0, StataCorp LLC, College Station, TX, USA) for multinomial regression models, and via SPSS for mediation analysis.

## 3. Results

### 3.1. Descriptions of Participants’ Characteristics

The current study included 11,031 participants, of which 54.37% (*n* = 5998) were females, 27.98% (*n* = 3087) were adolescents and the young, and 61.62% (*n* = 6797) were middle-aged. Over two-thirds of the participants (*n* = 7400, 67.08%) reported daily mobile phone use, and more than half (*n* = 5973, 54.15%) used a computer no more than three days per week. Descriptions of participants’ characteristics are provided in Table 1, and age- and sex-specific descriptions are shown in Table S2.

The mental health status of participants stratified by age and sex is shown in Figure 1 and Table S3. 19.99% of participants (*n* = 2205) reported the presence of comorbid mental health symptoms (two or three), with the highest prevalence observed in adolescents and young people (*n* = 728; 23.58%, 95% CI: 22.08–25.08%), followed by middle-aged group (18.88%, *n* = 1283, 95% CI: 17.95–19.81%) and older people (16.91%, *n* = 194, 95% CI: 14.74–19.07%). Characteristic-specific comorbid mental health symptoms in the three age groups are displayed in Tables S4 and S5.

### 3.2. Contribution of Electronic Device Use to Comorbid Mental Health Symptoms

The results of predictive feature importance for comorbid mental health symptoms in different age and sex groups are displayed in Figure 2 (comorbidity vs. no) and Figure S1 (one mental health symptom vs. no), and the corresponding ROC curves and AUC values are presented in Figures S2 and S3, while model accuracy indices are detailed in Table S6. There were notable variations across age and sex groups: eating behavior contributed the most to comorbid mental health symptoms in all age and sex groups; the weekly frequency of mobile phone use was the second most important predictor of comorbid mental health symptoms in adolescents and young people as well as in middle-aged people, while it was fourth in older males. The contribution of weekly frequency of computer use was significant in all males and older females. It should be noted that the random forest model did not present a high level of accuracy, and the related outcomes should be considered exploratory.

### 3.3. Associations Between Electronic Device Use and Comorbid Mental Health Symptoms

After fitting the multinomial regression model and adjusting for covariates, we found that high-frequency mobile phone use was negatively associated with comorbid mental health symptoms in all three age groups (adolescents and young people, OR = 0.38, 95% CI: 0.18–0.78; middle-aged people, OR = 0.36, 95% CI: 0.20–0.65; older people, OR = 0.37, 95% CI: 0.20–0.69), while the association was not statistically significant with high-frequency computer use. Low- and mid-frequency computer use was positively correlated with comorbid mental health symptoms in both middle-aged people (OR = 1.63, 95% CI: 1.25–2.11 vs. OR = 1.48, 95% CI: 1.11–1.97) and older people (OR = 2.69, 95% CI: 1.58–4.60 vs. OR = 3.43, 95% CI: 1.62–7.29). The results of multinomial regression are shown in Table S7.

The subgroup analyses by sex indicated that a significantly negative association between high frequency of mobile phone use and comorbid mental health symptoms was noted in young and middle-aged males, but not in females in the same age groups. The positive association with low- and mid-frequency computer use was significant in males of all age groups (Figure 3 and Table S8).

### 3.4. The Role of Self-Efficacy and Social Support

By fitting the SEM with the adjustment for covariates, regarding the weekly frequency of mobile phone use, the models revealed that there was no significant direct association of weekly frequency of mobile phone use with comorbid mental health outcomes for both sexes. In males, social support and self-efficacy played significant mediating roles in the relationships between mid- and high-frequency use and comorbidity in adolescents and young people as well as older people, with significant effects in relation to all different frequencies of use among middle-aged people. In females, the indirect role of social support was not significant in older people. The model parameters for mobile phone use are shown in Figure 4.

With respect to the weekly frequency of computer use (Figure S4), the significant direct association with comorbid mental health outcomes was identified in middle-aged people of both sexes and in older males. Social support and self-efficacy played significant mediating roles in the relationships between mid- and high-frequency use and comorbidity in adolescents and young people and middle-aged people of both sexes. The role of social support was not significant in older females, while self-efficacy presented significant roles in the relationship between high-frequency use and comorbidity in older adults of both sexes.

## 4. Discussion

Taking advantage of a large-scale nationally representative survey encompassing 120 cities across 32 provinces in China, this study elucidates the intricate relationships between the weekly frequency of electronic device use and comorbid mental health symptoms across age and sex groups. Although there was no significant direct association of the weekly frequency of mobile phone use with comorbid mental health outcomes in both sexes, and the direct role of weekly frequency of computer use was only noted in middle-aged and older males, the mediating roles of self-efficacy and social support were significant, which supports H2. The results revealed nuanced patterns, underscoring the necessity for a multidimensional approach to understanding the roles of mobile phones and computers in mental well-being.

The weekly frequency of mobile phone use presented a negative association with comorbid mental health symptoms, especially among male adolescents and young males, which validated H1 and was similar to the findings of the previous study indicating that young adult students without a mobile phone showed a higher likelihood of presenting anxiety symptoms, depressive symptoms and stress to a lesser extent [61]. While our empirical data do not directly test these underlying mechanisms, one plausible explanation for the negative association we observed is the social connectivity facilitated by mobile devices [62]. Although our study did not specify the purpose of device use, prior literature suggested that for adolescents, mobile phones primarily act as a preferred tool for accessing online studying resources, maintaining peer relationships [63], approaching online support communities, and engaging in stress-relieving activities (e.g., online games) [64], which are inversely associated with the adolescents’ mental health risks [61,65]. In addition, low-frequency computer use (1–3 days/week) was identified to be associated with higher odds of the presence of one mental health symptom among young males. While the present study did not stratify computer use by specific purpose, one potential explanation is that this limited computer engagement is primarily tied to academic or occupational demands [66], which may exacerbate stress in contexts requiring digital competence [30]. It should be noted that previous studies found a significant association between excessive screen time and anxiety and depression in youth [67,68], while screen time was not available in the current dataset, limiting the further exploration of this perspective. We also observed the significant mediating roles of self-efficacy and social support in the relationship of interest in this age group. Previous literature suggested that moderate mobile phone use might support adolescents’ well-being, which may partially explain our findings. Other studies have indicated that online games were associated with affective well-being [69,70] and life satisfaction [64], especially in male adolescents, which might relate to increased self-efficacy [71] and better mental health in adolescents, suggesting that interventions targeting or benefitting from these factors should be age-tailored. Notably, these inverse relationships warrant cautious interpretation. Specifically, individuals with better mental health, higher social engagement, and greater self-efficacy may be more likely to use mobile devices to sustain social connections. In contrast, individuals experiencing elevated anxiety or depressive symptoms tend to disengage from digital communication and online social networks [9,72]. Accordingly, pre-existing mental well-being may serve as a driver to shape healthy digital behavioral patterns, indicating that the observed association could be bidirectional or mutually reinforcing rather than unidirectionally protective.

Middle-aged populations exhibited divergent patterns based on device type, i.e., moderate weekly frequency of computer use (4–5 days/week) was significantly associated with higher odds of comorbid mental health symptoms, particularly among males. A previous study in the occupational field has identified prolonged computer use as a source of technostress [73], an association particularly plausible among middle-aged populations, for whom computer use is predominantly shaped by high-intensity work demands [2]. Notably, this study was limited by a lack of data on the specific purposes of usage, precluding differentiation between work-related and recreational device activities. Consequently, the observed associations between frequent computer use and adverse mental health outcomes may not be solely attributable to occupational technostress but could also reflect co-occurring behavioral correlates of prolonged device exposure, including sedentary lifestyles and reduced physical socialization [74]. Future investigations incorporating detailed categorization of screen-based activities are warranted to disentangle the distinct mental health impacts of work-induced technostress and lifestyle-related risk factors. Middle-aged individuals shoulder significant familial and financial responsibilities, and excessive device use related to work could disrupt restorative activities [75] and cause sleep disturbances [76]. In contrast, those with a high frequency of mobile phone use were less likely to present mental health comorbidity in this group, likely due to the mobile phone’s role in sustaining social and professional networks. Mobile phones facilitate flexible communication, help to alleviate the work–family conflict that is one of the common stressors in middle adulthood, which was also examined in the current study by identifying the prominent mediating role of social support, highlighting its importance in mitigating digital work-related stressors [26,77].

Sex disparities in the relationships of interest were evident among different age groups. A significant negative association between high-frequency mobile phone use and mental health symptoms was observed in young males, likely due to online gaming and peer interactions that foster a sense of competence and belonging [78]. There was no significant association between digital device use and mental health symptoms in female adolescents and young females, but frequent mobile phone use was negatively associated with comorbid mental health symptoms among older females. Digital engagement is positively related to the well-being of aging populations by enhancing social participation and accessing health information [10,79], especially for older females; mobile phones could enable intergenerational communication and access to online communities, which is advantageous in countering loneliness, a key driver of late-life depression [80]. For middle-aged males, the dual burden of occupational computer use and familial responsibilities may partially explain the elevated mental health risks [81]. Interventions promoting workplace flexibility [82] and improving psychological capital [83] may be considered in practice as potential solutions. However, low- or mid-frequency computer use (1–5 days/week) was associated with increased mental health comorbidities among older males, which could relate to generational gaps in digital literacy. Unlike mobile phones, operating a computer often demands more complex interactions, which might pose challenges for older males with lower self-efficacy, potentially leading to frustration.

Consistent with H2, we found that self-efficacy and social support mediated the relationships between the frequency of electronic device use and comorbid mental health symptoms, with variations by age and sex. Among adolescents and the young, high self-efficacy alleviated the stress of balancing academic demands and digital engagement [30,84], while social support provided protection against cyberbullying or online harassment [78]. For the middle-aged, social support from family and colleagues mediated the associations between the frequency of mobile phone use and mental health symptoms, while low self-efficacy intensified computer-related stress [85]. Notably, these mediators showed limited associations with mental health symptoms in older people, suggesting that factors such as physical health or socioeconomic status may play a significant role in mental health outcomes [29]. Complementary aspects such as digital literacy and accessibility of digital technology should be further investigated in the older groups.

Our study extended the existing literature by providing the age- and sex-specific relationships between the weekly frequency of computer and mobile phone use and comorbid mental health symptoms, and the roles of social support and self-efficacy. The present study provided theoretical insights by demonstrating that the potential buffering roles of self-efficacy and social support against digital-related mental health comorbidities are age- and sex-specific, rather than universally generalizable. Practically, these findings might provide stakeholders with specific evidence to inform stratified digital health governance. Specifically, policymakers should shift from uniform device-use restrictions to age- and sex-adapted strategies. For occupational settings, institutions could adopt targeted workplace flexibility arrangements and psychological capital training to mitigate computer-related technostress in middle-aged populations.

However, there are some limitations that should be acknowledged. First, the cross-sectional design precludes causal inferences between exposures and outcomes. Second, the present study quantified electronic device exposure based on weekly use frequency, without capturing the actual volume, intensity, duration, or functional purpose. Therefore, weekly frequency cannot be equated with total screen time. Although this frequency-based metric captured the regularity and habitual nature of device access, it failed to quantify the magnitude and distinguish between active and passive digital engagement. Such measurement ambiguity may mask critical dose–response thresholds separating adaptive digital engagement from maladaptive, health-impairing use and constrain nuanced interpretation of behavior-specific associations, including those related to gaming, occupational device use, and technostress. Future studies should integrate comprehensive, multidimensional exposure metrics to enable precise characterization of technology use and clarify its mental health implications. Third, mental health outcomes were evaluated using the self-reported GAD-7, PHQ-9, and PSS-3 questionnaires, which may introduce self-report bias. Accordingly, the present findings capture subclinical symptomatic presentations rather than clinically diagnosed mental disorders. Fourth, the extensive age and sex subgroup analyses may introduce a risk of multiple testing. Since the stratified models in our study were designed to identify subgroup-specific association patterns, differences in significance levels between demographic groups should therefore be interpreted as exploratory rather than confirming definitive between-group differences. Finally, despite comprehensive analytical adjustments implemented to optimize model performance, the RF model did not present a high level of predictive accuracy, which means that the results should be interpreted as exploratory and further validated.

## 5. Conclusions

In conclusion, using data from a large-scale Chinese population, this study provides comprehensive evidence by specifically indicating that a high weekly frequency of mobile phone use was negatively associated with the presence of comorbid mental health symptoms in all three age groups, especially in young and middle-aged males, and that a positive association with low- and mid-frequency computer use was significant in males from all age groups. Self-efficacy and social support differentially mediated these relationships across age and sex groups. The implementation of technology-driven strategies, such as moderated peer-support mobile applications or gamified mental health platforms tailored for young males, should be considered to foster digital support networks. In addition, community practitioners should prioritize the enhancement of digital inclusion and literacy initiatives for older adults. Given the cross-sectional design and reliance on self-reported weekly measurements, the practical implications of this study should be interpreted within contextual constraints, and further longitudinal research should be conducted to confirm causality.

## Figures and Tables

**Figure 1:**
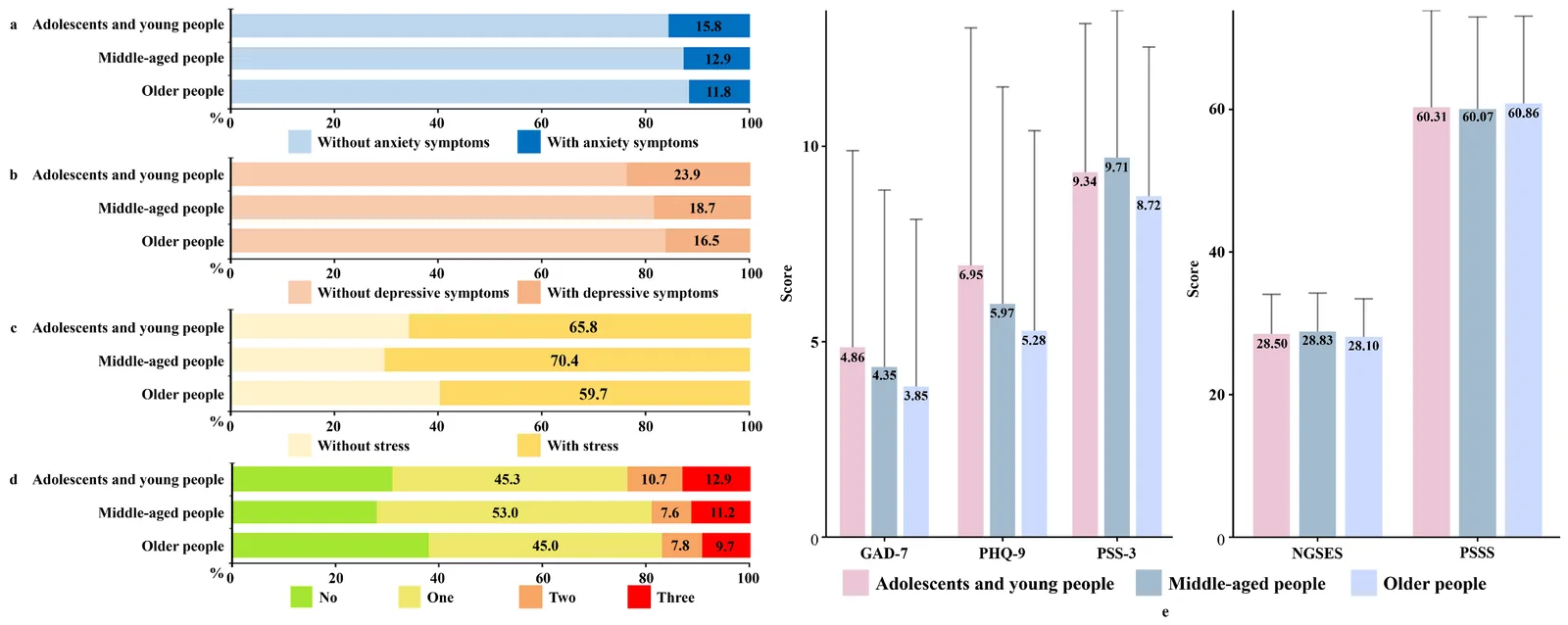
Mental health status of participants in different age groups: (**a**) anxiety symptoms (%), (**b**) depressive symptoms (%), (**c**) stress (%), (**d**) number of mental health symptoms (%), (**e**) scores of mental health symptoms (mean ± SD). GAD-7: General Anxiety Disorder-7. PHQ-9: Patient Health Questionnaire. PSS-3: 3-item Perceived Stress Scale. NGSES: New General Self-Efficacy Scale. PSSS: Perceived Social Support Scale.

**Figure 2:**
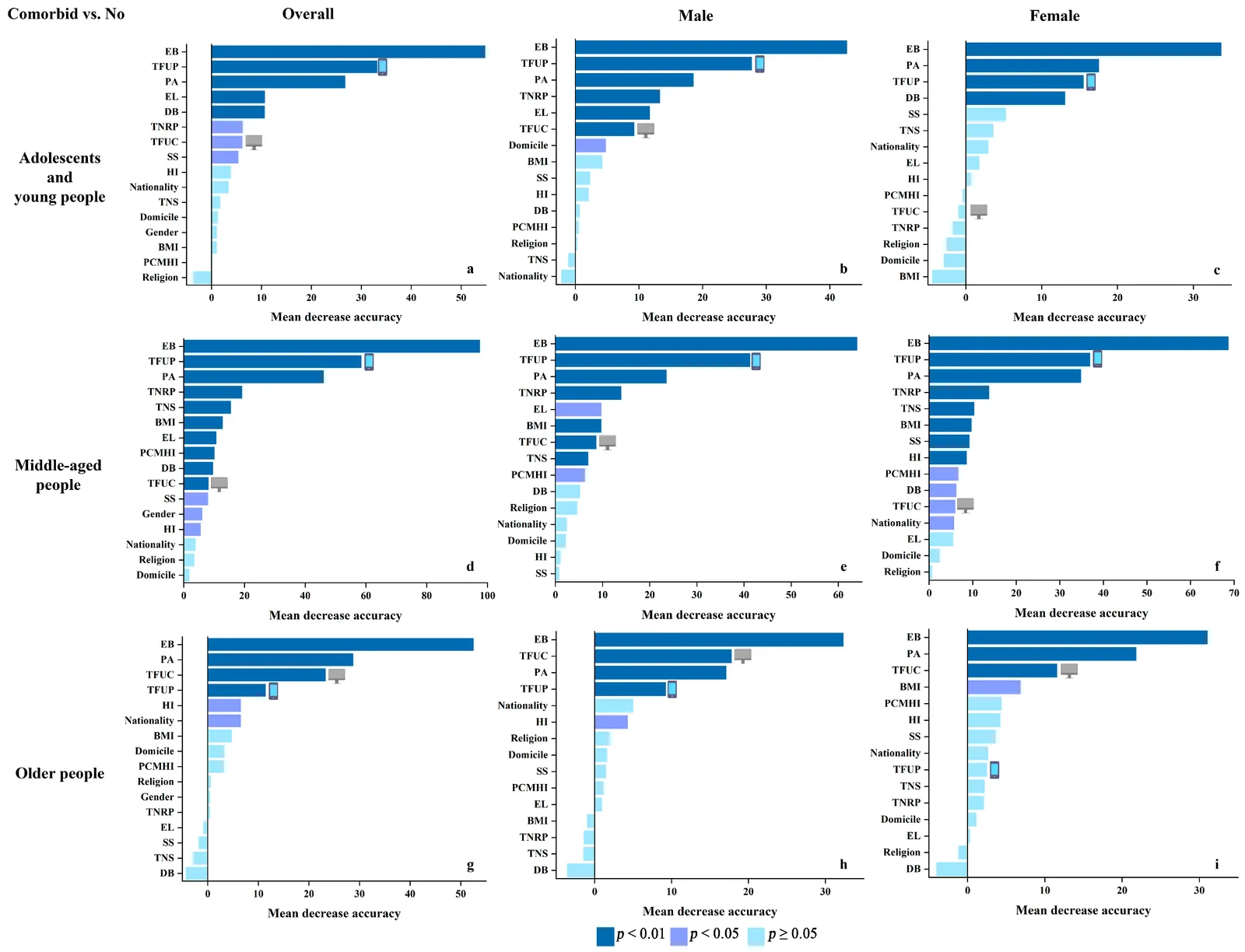
Feature importance for comorbid mental health symptoms in different age and sex groups from random forest algorithm (comorbid vs. no): (**a**) Overall adolescents and young people; (**b**) male adolescents and young people; (**c**) female adolescents and young people; (**d**) overall middle-aged people; (**e**) middle-aged males; (**f**) middle-aged females; (**g**) overall older people; (**h**) older males; (**i**) older females. RA: residential areas. RB: religious belief. PA: physical activity. EB: eating behavior. TFCU: the frequency of computer use. TFMPU: the frequency of mobile phone use. MHIPC: monthly household income per capita. EL: educational level. HI: household indebtedness. TNRP: the number of residential properties owned by family. SS: smoking status. AC: alcohol consumption. TNS: the number of siblings.

**Figure 3:**
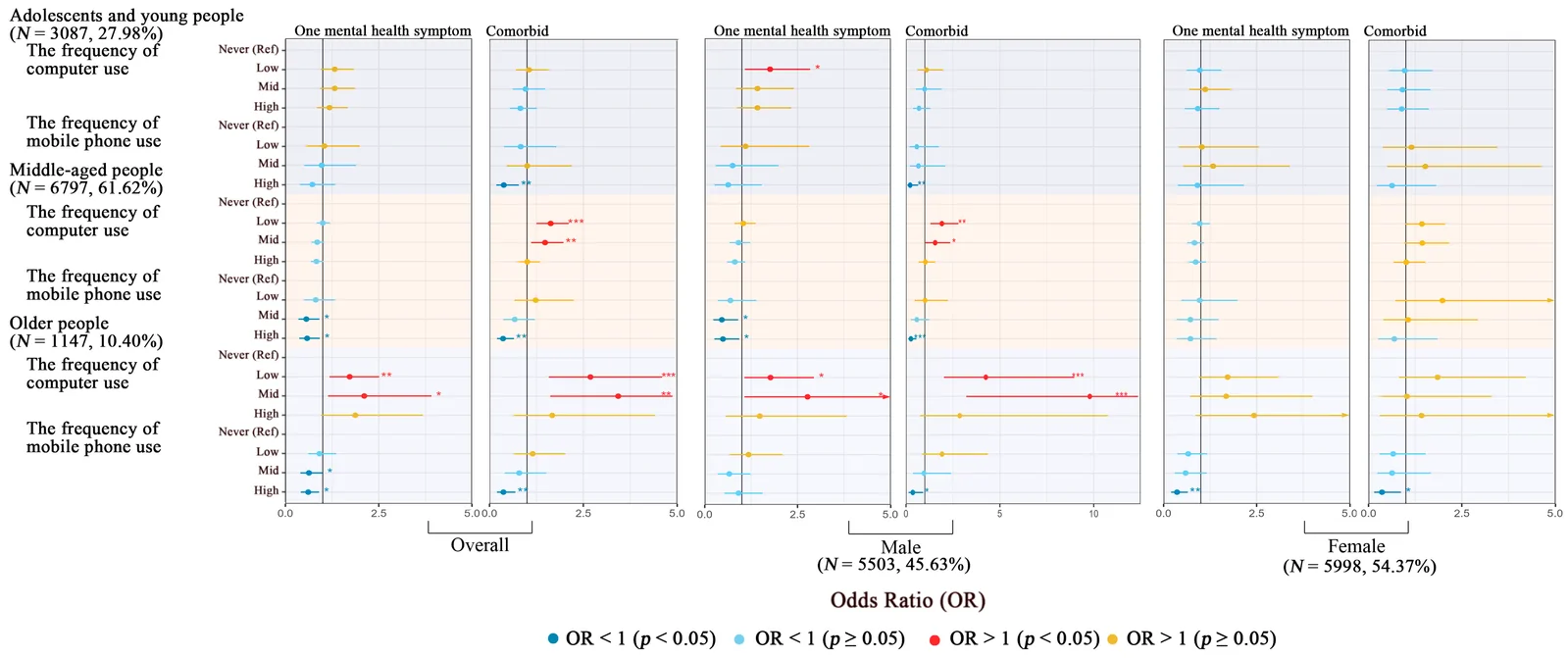
The association between the frequency of electronic device use and comorbid mental health symptoms in different age and sex groups. Reference group: No mental health symptoms. Adjusted by nationality, religious belief, educational level, residential areas, the number of residential properties owned by family, household indebtedness, monthly household income per capita, number of siblings, smoking status, alcohol consumption, BMI, physical activity, and eating behavior, as well as sex for only overall groups. Arrows represent truncated confidence intervals beyond the axis limits. * *p* < 0.05, ** *p* < 0.01, *** *p* < 0.001.

**Figure 4:**
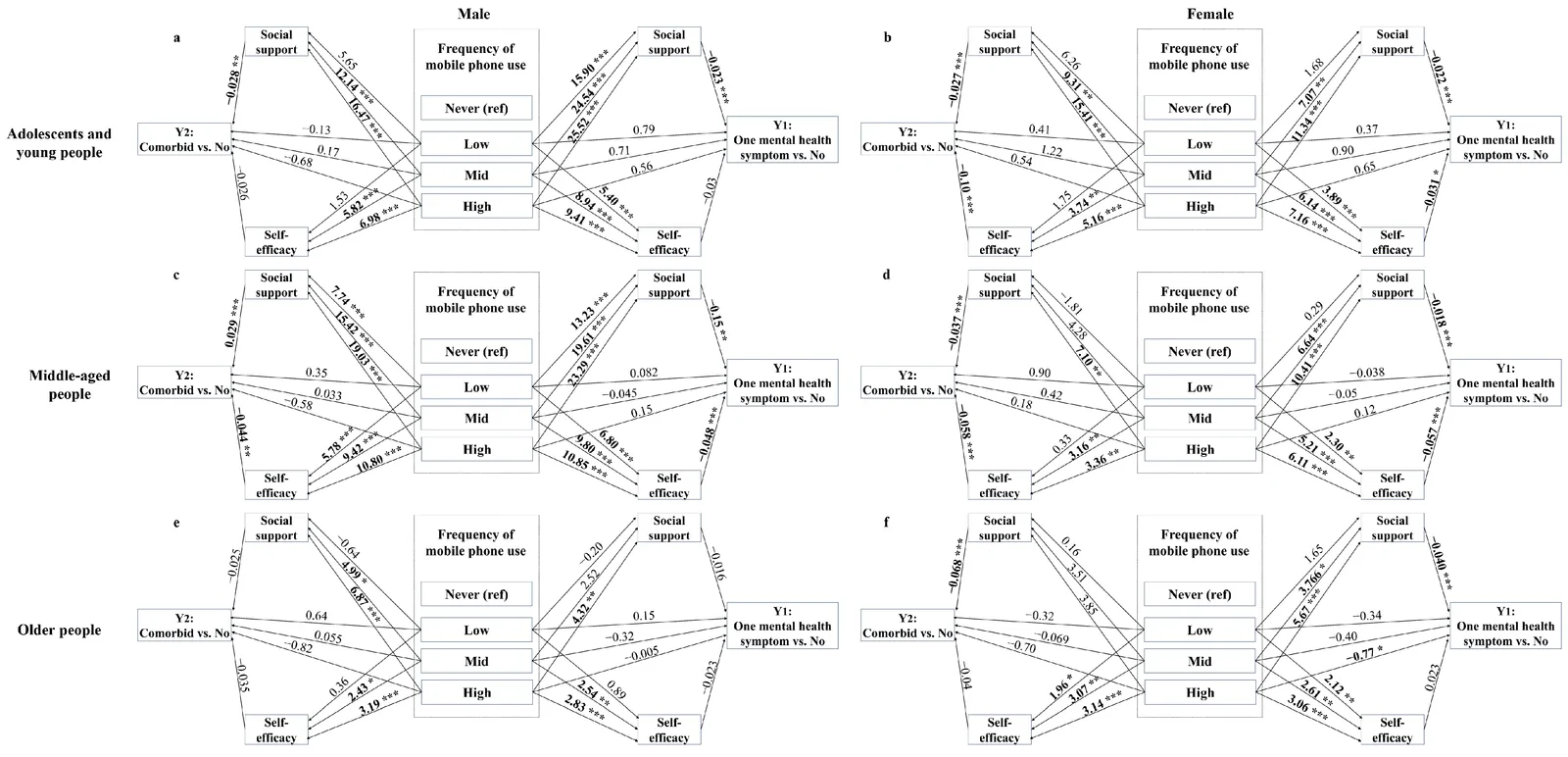
The mediating roles of self-efficacy and social support in the relationship between the weekly frequency of mobile phone use and comorbid mental health symptoms: (**a**) Male adolescents and young people; (**b**) female adolescents and young people; (**c**) middle-aged males; (**d**) middle-aged females; (**e**) older males; (**f**) older females. Adjusted by nationality, religious belief, educational level, residential areas, the number of residential properties owned by family, household indebtedness, monthly household income per capita, number of siblings, smoking status, alcohol consumption, BMI, physical activity, and eating behavior. * *p* < 0.05, ** *p* < 0.01, *** *p* < 0.001.

**Table 1:** Characteristics of participants by age group.

Characteristics	Overall (*N* = 11,031)	Adolescents and Young People (*N* = 3087, 27.98%)	Middle-Aged People (*N* = 6797, 61.62%)	Older People (*N* = 1147, 10.40%)	*F*/X^2^ Value (*p*-Value)
**Sex**					27.57 (<0.001)
Male	5033 (45.63)	1303 (42.21)	3149 (46.33)	581 (50.65)	
Female	5998 (54.37)	1784 (57.79)	3648 (53.67)	566 (49.35)	
**Nationality**					5.04 (0.08)
The Han	10,386 (94.15)	2883 (93.39)	6425 (94.53)	1078 (93.98)	
Others	645 (5.85)	204 (6.61)	372 (5.47)	69 (6.02)	
**Religious belief**					16.95 (<0.001)
No	10,709 (97.08)	3022 (97.89)	6591 (96.97)	1096 (95.55)	
Yes	322 (2.92)	65 (3.11)	206 (3.03)	51 (4.45)	
**Educational level**					1903.5 (<0.001)
Primary school and below	1127 (10.22)	125 (4.05)	496 (7.30)	506 (44.11)	
Middle school	3417 (30.98)	790 (25.59)	2196 (32.31)	431 (37.58)	
College/university degree or above	6487 (58.80)	2172 (70.36)	4105 (60.39)	210 (18.31)	
**Residential areas**					201.76 (<0.001)
Urban	8008 (72.60)	2158 (69.91)	5196 (76.45)	654 (57.02)	
Rural	3023 (27.40)	929 (30.10)	1601 (23.55)	493 (42.98)	
**The number of residential properties owned by family**					55.44 (<0.001)
No	1083 (9.82)	352 (11.40)	641 (9.43)	90 (7.85)	
Only one	6598 (59.81)	1694 (54.88)	4145 (60.98)	759 (66.17)	
More than or equal to two	3350 (30.37)	1041 (33.72)	2011 (29.59)	298 (25.98)	
**Household indebtedness**					231.8 (<0.001)
No	6780 (61.46)	1785 (57.82)	4054 (59.64)	941 (82.04)	
Yes	4251 (38.54)	1302 (42.18)	2743 (40.36)	206 (17.96)	
**Monthly household income per capita (CNY)**					206.22 (<0.001)
Low (≤3000)	3246 (29.43)	1075 (34.82)	1700 (25.01)	471 (41.06)	
General (3001–6000)	4254 (38.56)	1103 (35.73)	2745 (40.39)	406 (35.40)	
Middle (6001–8999)	1860 (16.86)	452 (14.64)	1231 (18.11)	177 (15.43)	
High (≥9000)	1671 (15.15)	457 (14.80)	1121 (16.49)	93 (8.11)	
**Number of siblings**					496.92 (<0.001)
No	2564 (23.24)	1120 (36.28)	1360 (20.01)	84 (7.32)	
One or more	8467 (76.76)	1967 (63.72)	5437 (79.99)	1063 (92.68)	
**Smoking status**					247.16 (<0.001)
Non-smokers or ex-smokers	9632 (87.32)	2941 (95.27)	5709 (83.99)	982 (85.61)	
Current smokers	1399 (12.68)	146 (4.73)	1088 (16.01)	165 (14.39)	
**Alcohol consumption in the past year**					54.78 (<0.001)
No	6578 (59.63)	1903 (61.65)	3894 (57.29)	781 (68.09)	
Yes	4453 (40.37)	1184 (38.35)	2903 (42.71)	366 (31.91)	
**Physical activity**					168.53 (<0.001)
No (0)	724 (6.56)	164 (5.31)	439 (6.46)	121 (10.55)	
Moderate level (1–10)	7424 (67.30)	1966 (63.69)	4574 (67.29)	884 (77.07)	
High (11–24)	2883 (26.14)	957 (31.00)	1784 (26.25)	142 (12.38)	
**EBS-SF score, Mean (SD)**	16.45 (4.65)	17.41 (4.53)	16.38 (4.57)	14.32 (4.71)	355.9 (<0.001)
**Eating Behavior**					337.52 (<0.001)
Healthy (7–14)	3712 (33.65)	793 (25.69)	2292 (33.72)	627 (54.66)	
General (15–20)	4865 (44.10)	1443 (46.74)	3045 (44.80)	377 (32.87)	
Unhealthy (21–28)	2454 (22.25)	851 (27.57)	1460 (21.48)	143 (12.47)	
**BMI, Mean (SD)**	21.87 (3.18)	20.72 (3.10)	22.38 (3.09)	22.12 (3.17)	392.8 (<0.001)
**Frequency of computer use**					1618.9 (<0.001)
Never	2139 (19.39)	265 (8.58)	1171 (17.23)	703 (61.29)	
Low (1–3 days per week)	3834 (34.76)	1089 (35.28)	2457 (36.15)	288 (25.11)	
Mid (4–5 days per week)	2348 (21.29)	759 (24.59)	1492 (21.95)	97 (8.46)	
High (6–7 days per week)	2710 (24.57)	974 (31.55)	1677 (24.67)	59 (5.14)	
**Frequency of mobile phone use**					1796.3 (<0.001)
Never	536 (4.86)	68 (2.20)	146 (2.15)	322 (28.07)	
Low (1–3 days per week)	1663 (15.08)	471 (15.26)	896 (13.18)	296 (25.81)	
Mid (4–5 days per week)	1432 (12.98)	341 (11.05)	911 (13.40)	180 (15.69)	
High (6–7 days per week)	7400 (67.08)	2207 (71.49)	4844 (71.27)	349 (30.43)	

Notes: EBS-SF: Short-form Sakata Eating Behavior Scale. Chinese Yuan: CNY.

## Data Availability

Data used in this study are from the 2021 Psychology and Behavior Investigation of Chinese Residents (PBICR), which is available upon request at https://www.x-mol.com/groups/pbicr (accessed on 27 September 2024). More information can be emailed to liser@outlook.com.

## Supplementary Materials

The following supporting information can be downloaded at: https://www.mdpi.com/article/10.3390/healthcare14193140/s1, Figure S1: Feature importance for one mental health symptom in different age and sex groups from random forest algorithm (one mental health symptom vs. no); Figure S2: ROC curves and AUC values for comorbid mental health symptoms in different age and sex groups from random forest algorithm (comorbid vs. no); Figure S3: ROC curves and AUC values for one mental health symptom in different age and sex groups from random forest algorithm (one mental health symptom vs. no); Figure S4: The mediating effects of self-efficacy and social support in the relationship between the weekly frequency of computer use and comorbid mental health symptoms; Table S1: Regions of participants; Table S2: Characteristics of respondents by age and sex groups; Table S3: Mental health status of respondents by age and sex groups; Table S4: Characteristics of respondents by mental health status (overall); Table S5: Characteristics of respondents by mental health status and age group; Table S6: Accuracy of random forest models in different age and sex groups; Table S7: The association between electronic device use and comorbid mental health symptoms in different age groups; Table S8: The association between electronic device use and comorbid mental health symptoms in different age and sex groups.

## Abbreviations

The following abbreviations are used in this manuscript:

PBICR	Psychology and Behavior Investigation of Chinese Residents
CNY	Chinese Yuan
BMI	Body Mass Index
CDSMS	Chronic Disease Self-Management Study Measures
GAD-7	Generalized Anxiety Disorder 7-item Scale
PHQ-9	Patient Health Questionnaire 9-item Scale
PSS-3	3-item Perceived Stress Scale
PSSS	12-item Self-reported Perceived Social Support Scale
NGSES	8-item New General Self-Efficacy Scale
SD	Standard Deviation
ANOVA	Analysis of Variance
RF	Random Forest
ROC	Receiver Operating Characteristic
AUC	Area Under the Curve
OR	Odds Ratio
CI	Confidence Interval
SEM	Structural Equation Model
