# Supplementary material for: Heterogeneities by Age and Sex in Associations Between Electronic Device Use Frequency and Comorbid Mental Health Symptoms: The Roles of Self-Efficacy and Social Support
Source: Healthcare (Basel). 2026 Sep 22;14(19):3140. doi: 10.3390/healthcare14193140 (PMC13665081; doi:10.3390/healthcare14193140)
Supplement: Supplementary file 1 [file healthcare-14-03140-s001.zip › healthcare-4526027-supplementary.pdf]

Heterogeneities by Age and Sex in Associations Between Electronic Device Use Frequency and Comorbid Mental Health Symptoms: The Roles of Self-Efficacy and Social Support

Supplementary Information

**Figure S1**	Feature importance for one mental health symptom in different age and sex groups from random forest algorithm (one mental health symptom vs. no)
**Figure S2**	ROC curves and AUC values for comorbid mental health symptoms in different age and sex groups from random forest algorithm (comorbid vs. no)
**Figure S3**	ROC curves and AUC values for one mental health symptom in different age and sex groups from random forest algorithm (one mental health symptom vs. no)
**Figure S4**	The mediating roles of self-efficacy and social support in the relationship between the weekly frequency of computer use and comorbid mental health symptoms
**Table S1**	Regions of participants
**Table S2**	Characteristics of respondents by age and sex groups
**Table S3**	Mental health status of respondents by age and sex groups
**Table S4**	Characteristics of respondents by mental health status (overall)
**Table S5**	Characteristics of respondents by mental health status and age group
**Table S6**	Accuracy of random forest models in different age and sex groups
**Table S7**	The association between electronic device use and comorbid mental health symptoms in different age groups
**Table S8**	The association between electronic device use and comorbid mental health symptoms in different age and sex groups

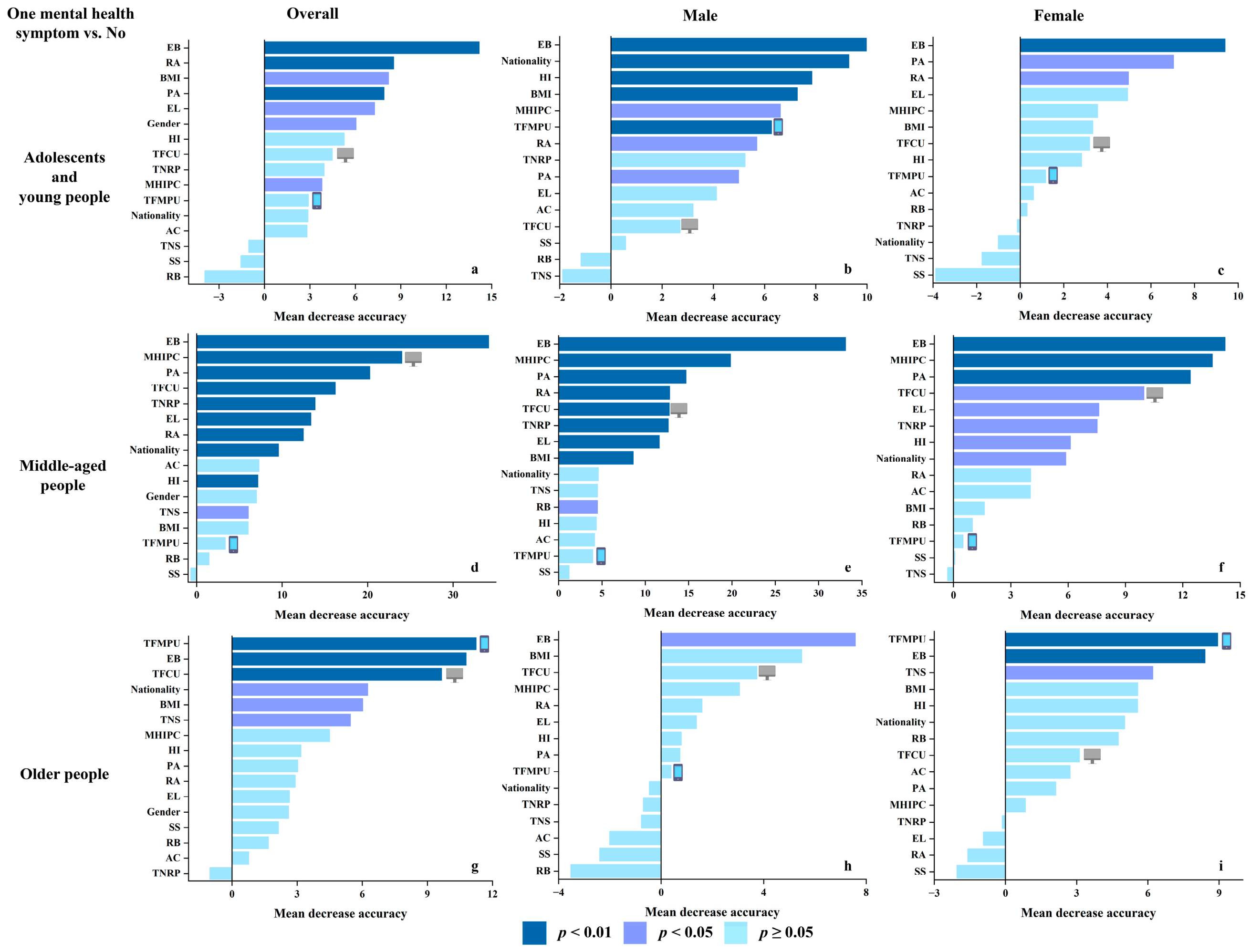

Figure S1. Feature importance for one mental health symptom in different age and sex groups from random forest algorithm (one mental health symptom vs. no): (**a**) overall adolescents and young people; (**b**) male adolescents and young people; (**c**) female adolescents and young people; (**d**) overall middle-aged people; (**e**) middle-aged males; (**f**) middle-aged females; (**g**) overall older people; (**h**) older males; (**i**) older females. RA: residential areas, RB: religious belief, PA: physical activity, EB: eating behavior, TFCU: the frequency of computer use, TFMPU: the frequency of mobile phone use, MHIPC: monthly household income per capita, EL: educational level, HI: household indebtedness, TNRP: the number of residential properties owned by family, SS: smoking status, AC: alcohol consumption, TNS: the number of siblings.

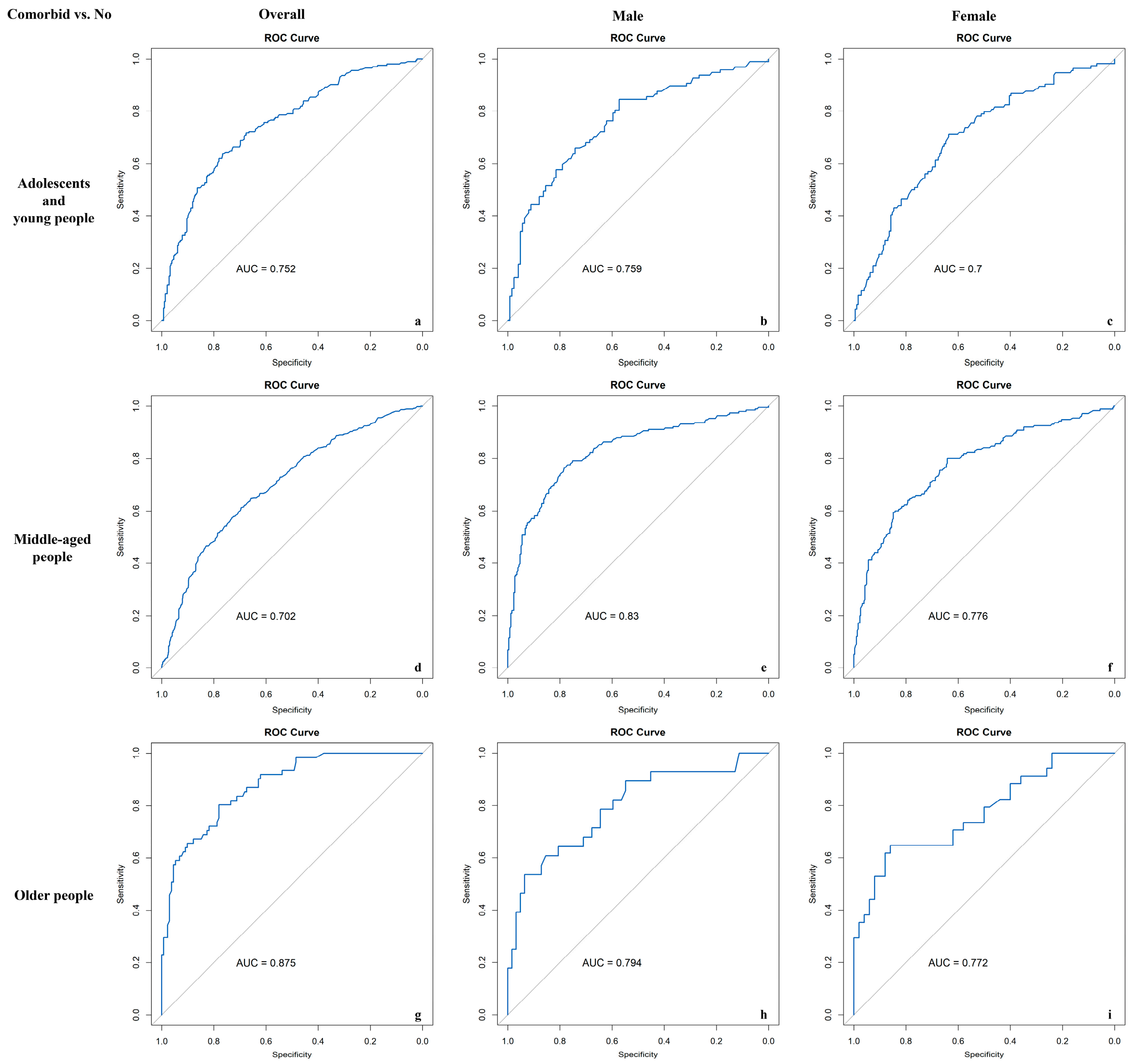

Figure S2. ROC curves and AUC values for comorbid mental health symptoms in different age and sex groups from random forest algorithm (comorbid vs. no): (**a**) overall adolescents and young people; (**b**) male adolescents and young people; (**c**) female adolescents and young people; (**d**) overall middle-aged people; (**e**) middle-aged males; (**f**) middle-aged females; (**g**) overall older people; (**h**) older males; (**i**) older females. ROC: Receiver Operating Characteristic; AUC: Area Under the Curve.

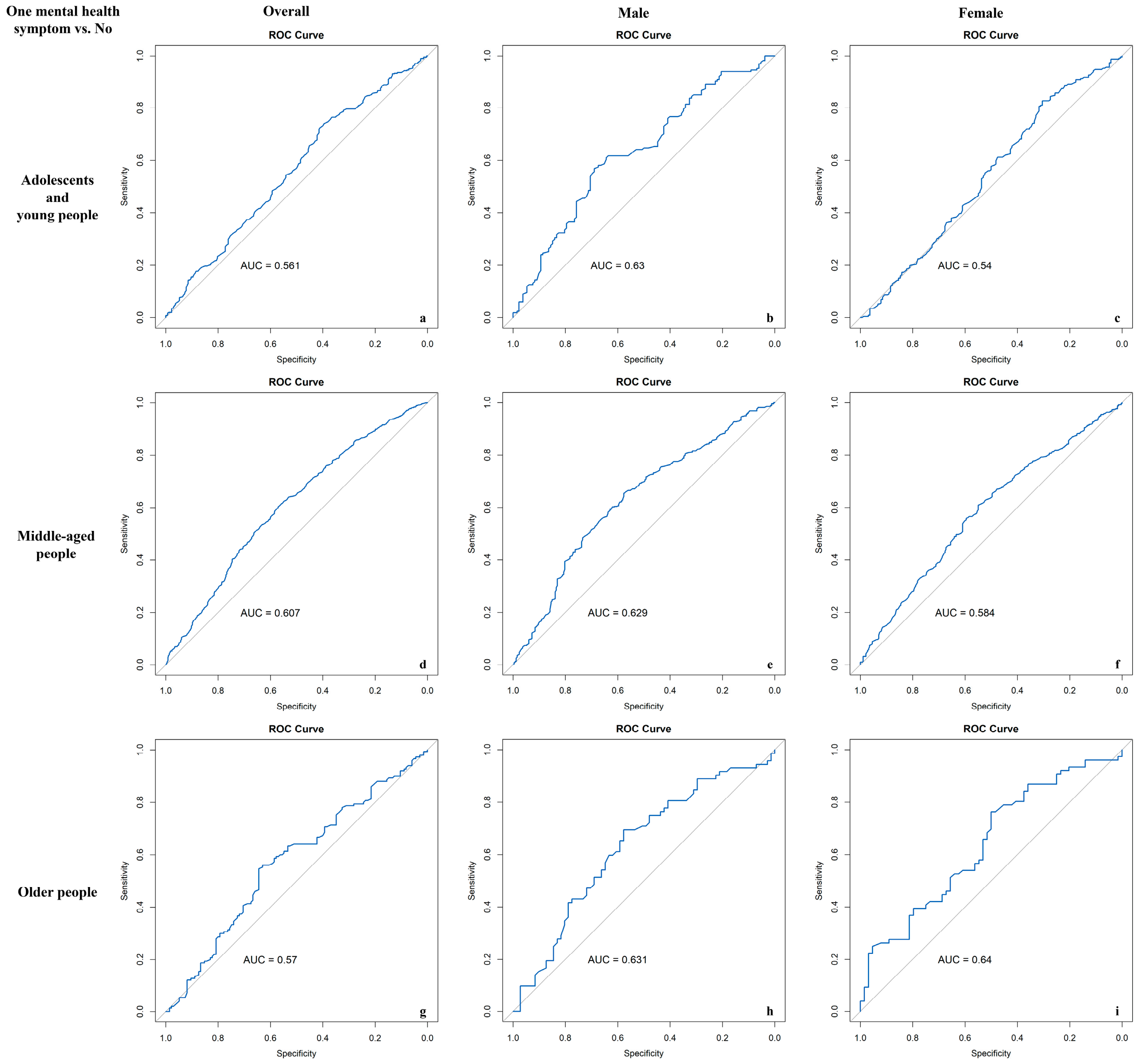

Figure S3. ROC curves and AUC values for one mental health symptom in different age and sex groups from random forest algorithm (one mental health symptom vs. no): (**a**) overall adolescents and young people; (**b**) male adolescents and young people; (**c**) female adolescents and young people; (**d**) overall middle-aged people; (**e**) middle-aged males; (**f**) middle-aged females; (**g**) overall older people; (**h**) older males; (**i**) older females. ROC: Receiver Operating Characteristic; AUC: Area Under the Curve.

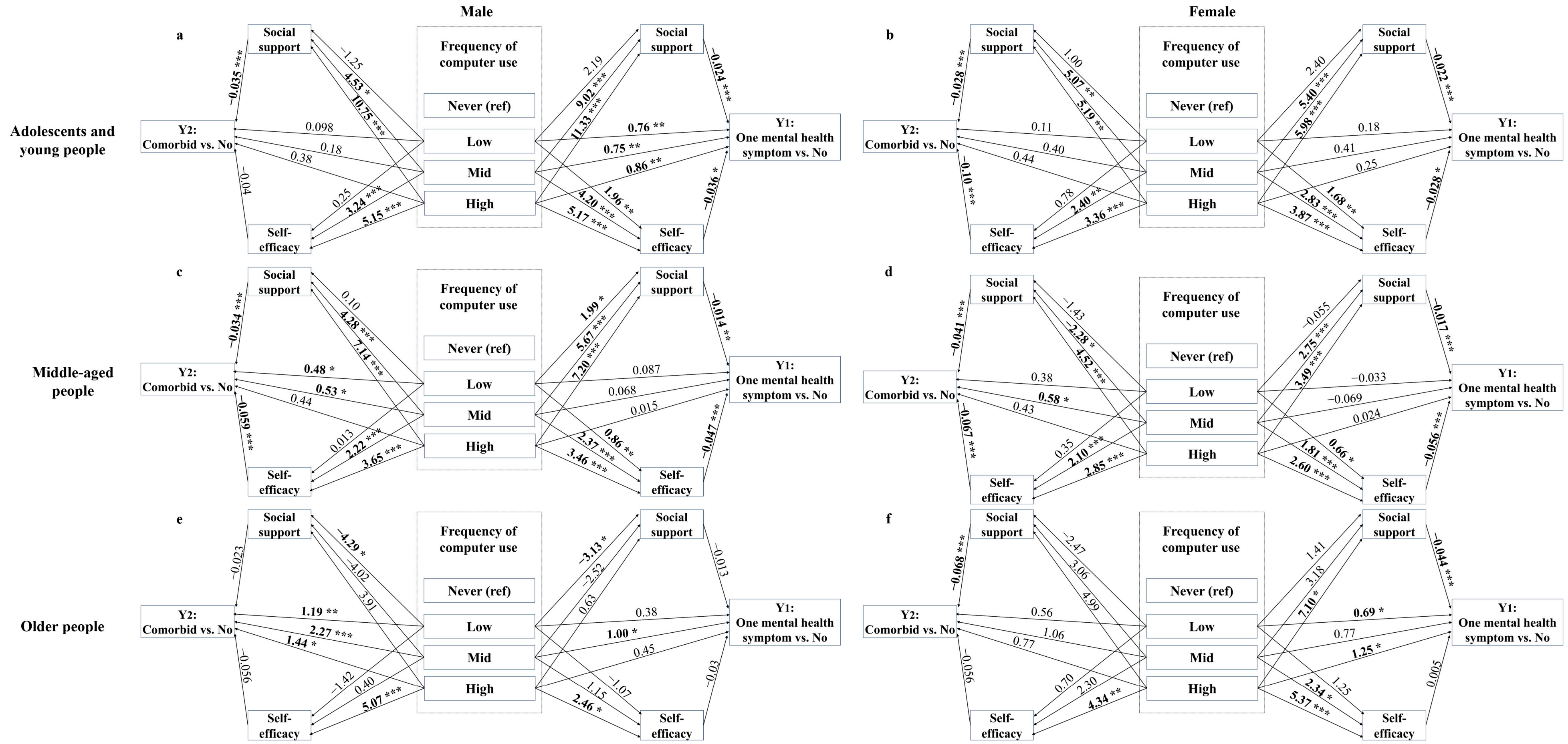

Figure S4. The mediating roles of self-efficacy and social support in the relationship between the weekly frequency of computer use and comorbid mental health symptoms: (**a**) male adolescents and young people; (**b**) female adolescents and young people; (**c**) middle-aged males; (**d**) middle-aged females; (**e**) older males; (**f**) older females. Adjusted by nationality, religious belief, educational level, residential areas, the number of residential properties owned by family, household indebtedness, monthly household income per capita, number of siblings, smoking status, alcohol consumption, BMI, physical activity, and eating behavior. * $p < 0.05$, ** $p < 0.01$, *** $p < 0.001$.

Table S1. Residing regions of participants

Provinces, autonomous regions and municipalities	*N* (%)	Provinces, autonomous regions and municipalities	*N* (%)
Beijing	472 (4.28)	Hubei	347 (3.15)
Tianjin	586 (5.31)	Hunan	250 (2.27)
Hebei	380 (3.44)	Guangdong	394 (3.57)
Shanxi	718 (6.51)	Guangxi	91 (0.82)
Inner Mongolia	136 (1.23)	Hainan	117 (1.06)
Liaoning	142 (1.29)	Chongqing	294 (2.67)
Jilin	118 (1.07)	Sichuan	298 (2.70)
Heilongjiang	73 (0.66)	Guizhou	246 (2.23)
Shanghai	66 (0.6)	Yunnan	267 (2.42)
Jiangsu	211 (1.91)	Tibet	16 (0.15)
Zhejiang	302 (2.74)	Shaanxi	422 (3.83)
Anhui	651 (5.9)	Gansu	54 (0.49)
Fujian	113 (1.02)	Qinghai	65 (0.59)
Jiangxi	284 (2.57)	Ningxia	246 (2.23)
Shandong	2827 (25.63)	Xinjiang	432 (3.92)
Henan	410 (3.72)	Taiwan	1 (0.01)

Note: There are two participants without location information.

Table S2. Characteristics of respondents by age and sex groups

Characteristics	Overall (N = 11,031)	Adolescents and young people (N = 3087, 27.98%)		Middle-aged people (N = 6797, 61.62%)		Older people (N = 1147, 10.40%)	
		Male (n = 1303, 42.21%)	Female (n = 1784, 57.79%)	Male (n = 3149, 46.33%)	Female (n = 3648, 53.67%)	Male (n = 581, 50.65%)	Female (n = 566, 49.35%)
**Nationality**							
The Han	10,386 (94.15)	1217 (93.40)	1666 (93.39)	2988 (94.89)	3437 (94.22)	549 (94.49)	529 (93.46)
Others	645 (5.85)	86 (6.60)	118 (6.61)	161 (5.11)	211 (5.78)	32 (5.51)	37 (6.54)
**Religious belief**							
No	10,709 (97.08)	1278 (98.08)	1744 (97.76)	3061 (97.21)	3530 (96.77)	569 (97.93)	527 (93.11)
Yes	322 (2.92)	25 (1.92)	40 (2.24)	88 (2.79)	118 (3.23)	12 (2.07)	39 (6.89)
**Education level**							
Primary school and below	1127 (10.22)	69 (5.30)	56 (3.14)	204 (6.48)	292 (8.00)	196 (33.73)	310 (54.77)
Middle school	3417 (30.98)	371 (28.47)	419 (23.49)	1066 (33.85)	1130 (30.98)	258 (44.41)	173 (30.57)
College/university degree or above	6487 (58.80)	863 (66.23)	1309 (73.37)	1879 (59.67)	2226 (61.02)	127 (21.86)	83 (14.66)
**Residential areas**							
Urban	8008 (72.60)	928 (71.22)	1230 (68.95)	2384 (75.71)	2812 (77.08)	335 (57.66)	319 (56.36)
Rural	3023 (27.40)	375 (28.78)	554 (31.05)	765 (24.29)	836 (22.92)	246 (42.34)	247 (43.64)
**The number of residential properties owned by family**							
No	1083 (9.82)	147 (11.28)	205 (11.49)	303 (9.62)	338 (9.27)	46 (7.92)	44 (7.77)
Only one	6598 (59.81)	714 (54.80)	980 (54.93)	1911 (60.69)	2234 (61.24)	383 (65.92)	376 (66.43)
More than or equal to two	3350 (30.37)	442 (33.92)	599 (33.58)	935 (29.69)	1076 (29.49)	152 (26.16)	146 (25.80)
**Household indebtedness**							
No	6780 (61.46)	775 (59.48)	1010 (56.61)	1884 (59.83)	2170 (59.48)	467 (80.38)	474 (83.75)
Yes	4251 (38.54)	528 (40.52)	774 (43.39)	1265 (40.17)	1478 (40.52)	114 (19.62)	92 (16.25)
**Monthly household income per capita (CNY)**							
Low (≤ 3000)	3246 (29.43)	369 (28.32)	706 (39.57)	735 (23.34)	965 (26.45)	232 (39.93)	239 (42.23)
General (3001–6000)	4254 (38.56)	490 (37.61)	613 (34.36)	1240 (39.38)	1505 (41.26)	210 (26.14)	196 (34.63)
Middle (6001–8999)	1860 (16.86)	201 (15.42)	251 (14.07)	605 (19.21)	626 (17.16)	83 (14.29)	94 (16.61)
High (≥ 9000)	1671 (15.15)	243 (18.65)	214 (12.00)	569 (18.07)	552 (15.13)	56 (9.64)	37 (6.54)
**Number of siblings**							
No	2564 (23.24)	544 (41.75)	576 (32.29)	715 (22.71)	645 (17.68)	46 (7.92)	38 (6.71)
One or more	8467 (76.76)	759 (58.25)	1208 (67.71)	2434 (77.29)	3003 (82.32)	535 (92.08)	528 (93.29)
**Smoking status**							
Non-smokers or ex-smokers	9632 (87.32)	1175 (90.18)	1766 (98.99)	2144 (68.09)	3565 (97.72)	429 (73.84)	553 (97.70)
Current smokers	1399 (12.68)	128 (9.82)	18 (1.01)	1005 (31.94)	83 (2.28)	152 (26.16)	13 (2.30)
**Alcohol consumption in the past year**							
No	6578 (59.63)	625 (47.97)	1278 (71.64)	1157 (36.74)	2737 (75.03)	299 (51.46)	482 (85.16)
Yes	4453 (40.37)	678 (52.03)	506 (28.36)	1992 (63.26)	911 (24.97)	282 (48.54)	84 (14.84)

**Physical activity**							
No (0)	724 (6.56)	70 (5.37)	94 (5.27)	226 (7.18)	213 (5.84)	44 (7.57)	77 (13.60)
Moderate level (1–10)	7424 (67.30)	715 (54.87)	1251 (70.12)	1920 (60.97)	2654 (72.75)	455 (78.31)	429 (75.80)
High (11–24)	2883 (26.14)	518 (39.76)	439 (24.61)	1003 (31.85)	781 (21.41)	82 (14.11)	60 (10.60)
**EBS-SF score, Mean (SD)**	16.45 (4.65)	17.43 (4.74)	17.39 (4.38)	16.52 (4.56)	16.25 (4.58)	14.38 (4.83)	14.27 (4.58)
**Eating Behavior**							
Healthy (7–14)	3712 (33.65)	346 (26.55)	447 (25.06)	1006 (31.95)	1286 (35.25)	311 (53.53)	316 (55.83)
General (15–20)	4865 (44.10)	581 (44.59)	862 (48.32)	1431 (45.44)	1614 (44.24)	194 (33.39)	183 (32.33)
Unhealthy (21–28)	2454 (22.25)	376 (28.86)	475 (26.63)	712 (22.61)	748 (20.50)	76 (13.08)	67 (11.84)
**BMI, Mean (SD)**	21.87 (3.18)	21.52 (3.32)	20.14 (2.79)	23.03 (3.15)	21.81 (2.91)	22.22 (15.39)	22.02 (3.33)
**Frequency of computer use**							
Never	2139 (19.39)	134 (10.28)	131 (7.34)	530 (16.83)	641 (17.57)	331 (56.97)	372 (65.72)
Low (1–3 days per week)	3834 (34.76)	436 (33.46)	653 (36.60)	1149 (36.49)	1308 (35.86)	167 (28.74)	121 (21.38)
Mid (4–5 days per week)	2348 (21.29)	266 (20.41)	493 (27.63)	688 (21.85)	804 (22.04)	50 (8.61)	47 (8.30)
High (6–7 days per week)	2710 (24.57)	467 (35.84)	507 (28.42)	782 (24.83)	895 (24.53)	33 (5.68)	26 (4.59)
**Frequency of mobile phone use**							
Never	536 (4.86)	36 (2.76)	32 (1.79)	91 (2.89)	55 (1.51)	136 (23.41)	186 (32.86)
Low (1–3 days per week)	1663 (15.08)	256 (19.65)	215 (12.05)	465 (14.77)	431 (11.81)	160 (27.54)	136 (24.03)
Mid (4–5 days per week)	1432 (12.98)	158 (12.13)	183 (10.26)	472 (14.99)	439 (12.03)	100 (17.21)	80 (14.13)
High (6–7 days per week)	7400 (67.08)	853 (65.46)	1354 (75.90)	2121 (67.35)	2723 (87.97)	185 (31.84)	164 (28.98)

Note: EBS-SF: Short-form Sakata Eating Behavior Scale. Chinese Yuan: CNY.

Table S3. Mental health status of respondents by age and sex groups

Mental health status	Overall (*N* = 11,031)	Adolescents and young people (*N* = 3087, 27.98%)			Middle-aged people (*N* = 6797, 61.62%)			Older people (*N* = 1147, 10.40%)		
		Overall	Male (*n* = 1303, 42.21%)	Female (*n* = 1,784, 57.79%)	Overall	Male (*n* = 3149, 46.33%)	Female (n = 3,648, 53.67%)	Overall	Male (*n* = 581, 50.65%)	Female (*n* = 566, 49.35%)
**Number of mental health symptoms**										
No	3306 (29.97)	960 (31.10)	407 (31.24)	553 (31.00)	1909 (28.09)	889 (28.23)	1020 (27.96)	437 (38.10)	230 (39.59)	207 (36.57)
Only one	5520 (50.04)	1399 (45.32)	582 (44.67)	817 (45.80)	3605 (53.04)	1619 (51.41)	1986 (54.44)	516 (44.99)	252 (43.37)	264 (46.64)
Two	937 (8.49)	329 (10.66)	131 (10.05)	198 (11.10)	519 (7.63)	245 (7.78)	274 (7.51)	89 (7.76)	47 (8.09)	42 (7.42)
Three	1268 (11.50)	399 (12.92)	183 (14.04)	216 (12.10)	764 (11.24)	396 (12.58)	368 (10.09)	105 (9.15)	52 (8.95)	53 (9.36)
**GAD-7 score, Mean (SD)**	4.44 (4.66)	4.86 (5.03)	4.93 (5.31)	4.81 (4.82)	4.35 (4.53)	4.44 (4.70)	4.28 (4.37)	3.85 (4.28)	3.64 (4.24)	4.07 (4.31)
**Anxiety symptoms**										
No (score < 10)	9534 (86.43)	2600 (84.22)	1078 (82.73)	1522 (85.31)	5922 (87.13)	2688 (85.36)	3234 (88.65)	1012 (88.23)	517 (88.98)	495 (87.46)
Yes (score ≥ 10)	1497 (13.57)	487 (15.78)	225 (17.27)	262 (14.69)	875 (12.87)	461 (14.64)	414 (11.35)	135 (11.77)	64 (11.02)	71 (12.54)
**PHQ-9 score, Mean (SD)**	6.18 (5.68)	6.95 (6.08)	6.94 (6.50)	6.96 (5.75)	5.97 (5.55)	6.09 (5.87)	5.87 (5.25)	5.28 (5.12)	5.13 (5.22)	5.44 (5.01)
**Depressive symptoms**										
No (score < 10)	8832 (80.07)	2350 (76.13)	983 (75.44)	1367 (76.63)	5524 (81.27)	2513 (79.80)	3011 (82.54)	958 (83.52)	484 (83.30)	474 (83.75)
Yes (score ≥ 10)	2199 (19.93)	737 (23.87)	320 (24.56)	417 (23.37)	1273 (18.73)	626 (20.20)	637 (17.46)	189 (16.48)	97 (16.70)	92 (16.25)
**PSS-3 score, Mean (SD)**	9.50 (3.79)	9.34 (3.80)	9.46 (4.09)	9.24 (3.58)	9.71 (3.77)	9.75 (3.84)	9.67 (3.70)	8.72 (3.82)	8.51 (3.83)	8.93 (3.80)
**Stress**										
No (0–7)	3529 (31.99)	1057 (34.24)	455 (34.92)	602 (33.74)	2010 (29.57)	949 (30.14)	1061 (29.08)	462 (40.28)	240 (41.31)	222 (39.22)
Yes (8–18)	7502 (68.01)	2030 (65.76)	848 (65.08)	1182 (66.26)	4787 (70.43)	2200 (69.86)	2587 (70.92)	685 (59.72)	341 (58.69)	344 (60.78)
**NGSES score, Mean (SD)**	28.66 (5.45)	28.50 (5.57)	28.43 (5.99)	28.55 (5.24)	28.83 (5.41)	28.61 (5.70)	29.02 (5.14)	28.10 (5.34)	28.48 (5.37)	27.71 (5.29)
**General Self Efficacy**										
Low (8–20)	538 (4.88)	166 (5.38)	87 (6.68)	79 (4.43)	311 (4.58)	176 (5.59)	135 (3.70)	61 (5.32)	22 (3.79)	39 (6.89)
Middle (21–30)	5645 (51.17)	1627 (52.70)	667 (51.19)	960 (53.81)	3361 (49.45)	1560 (49.54)	1801 (49.37)	657 (57.28)	325 (55.94)	332 (58.66)
High (31–40)	4848 (43.95)	1294 (41.92)	549 (42.13)	745 (41.76)	3125 (45.97)	1413 (44.87)	1712 (46.93)	429 (37.40)	234 (40.28)	195 (34.45)
**PSSS score, Mean (SD)**	60.22 (13.03)	60.31 (13.59)	59.50 (14.09)	60.89 (13.17)	60.07 (12.90)	59.04 (13.48)	60.96 (12.30)	60.86 (12.22)	60.39 (12.48)	61.35 (11.94)
**Social support**										
Low (12–36)	345 (3.13)	118 (3.82)	59 (4.53)	59 (3.31)	209 (3.07)	132 (4.19)	77 (2.11)	18 (1.57)	10 (1.72)	8 (1.41)
Middle (37–60)	5506 (49.91)	1490 (48.27)	642 (49.27)	848 (47.53)	3446 (50.70)	1658 (52.65)	1788 (49.01)	570 (49.69)	304 (52.53)	266 (47.00)
High (61–84)	5180 (46.96)	1479 (47.91)	602 (46.20)	877 (49.16)	3142 (46.23)	1359 (43.16)	1783 (48.88)	559 (48.74)	267 (45.96)	292 (51.59)

Note: GAD-7: Generalized Anxiety Disorder-7. PHQ-9: Patient Health Questionnaire-9items. PSS-3: 3-item Perceived Stress Scale. PSSS: Perceived Social Support Scale. NGSES: The New General Self-Efficacy Scale.

Table S4. Characteristics of respondents by mental health status (overall)

Characteristics	Overall (*N* = 11,031)	No mental health symptom (*N* = 3306, 29.97%)	Only one symptom (*N* = 5520, 50.04%)	Two or more symptoms (*N* = 2205, 19.99%)
**Age**				
Adolescents and young people (12–25)	3087 (27.98)	960 (29.04)	1399 (25.34)	728 (33.02)
Middle-aged people (26–59)	6797 (61.62)	1909 (57.74)	3605 (65.31)	1283 (58.19)
Older people (≥ 60)	1147 (10.40)	437 (13.22)	516 (9.35)	194 (8.79)
**Sex**				
Male	5033 (45.63)	1526 (46.16)	2453 (44.44)	1054 (47.80)
Female	5998 (54.37)	1780 (53.84)	3067 (55.56)	1151 (52.20)
**Nationality**				
The Han	10,386 (94.15)	3158 (95.52)	5172 (93.70)	2056 (93.24)
Others	645 (5.85)	148 (4.48)	348 (6.30)	149 (6.76)
**Religious belief**				
No	10,709 (97.08)	3222 (97.46)	5355 (97.01)	2132 (96.69)
Yes	322 (2.92)	84 (2.54)	165 (2.99)	73 (3.31)
**Educational level**				
Primary school and below	1127 (10.22)	350 (10.59)	550 (9.96)	227 (10.29)
Middle school	3417 (30.98)	989 (29.91)	1823 (33.03)	605 (27.44)
College/university degree or above	6487 (58.80)	1967 (59.50)	3147 (57.01)	1373 (62.27)
**Residential areas**				
Urban	8008 (72.60)	2419 (73.17)	4012 (72.68)	1577 (71.52)
Rural	3023 (27.40)	887 (26.83)	1508 (27.32)	628 (28.48)
**The number of residential properties owned by family**				
No	1083 (9.82)	235 (7.11)	494 (8.95)	354 (16.05)
Only one	6598 (59.81)	1939 (58.65)	3385 (61.32)	1274 (57.78)
More than or equal to two	3350 (30.37)	1132 (34.24)	1641 (29.73)	577 (26.17)
**Household indebtedness**				
No	6780 (61.46)	2125 (64.28)	3403 (61.65)	1252 (56.78)
Yes	4251 (38.54)	1181 (35.72)	2117 (38.35)	953 (43.22)
**Monthly household income per capita (CNY)**				
Low (≤ 3000)	3246 (29.43)	860 (26.01)	1658 (30.04)	728 (33.02)
General (3001–6000)	4254 (38.56)	1249 (37.78)	2225 (40.31)	780 (35.37)
Middle (6001–8999)	1860 (16.86)	641 (19.39)	855 (15.49)	364 (16.51)
High (≥ 9000)	1671 (15.15)	556 (16.82)	782 (14.16)	333 (15.10)
**Number of siblings**				
No	2564 (23.24)	738 (22.32)	1241 (22.48)	585 (26.53)
One or more	8467 (76.76)	2568 (77.68)	4279 (77.52)	1620 (73.47)
**Smoking status**				
Non-smokers or ex-smokers	9632 (87.32)	2917 (88.23)	4841 (87.70)	1874 (84.99)
Current smokers	1399 (12.68)	389 (11.77)	679 (12.30)	331 (15.01)
**Alcohol consumption in the past year**				
No	6578 (59.63)	2067 (62.52)	3339 (60.49)	1172 (53.15)

Yes	4453 (40.37)	1239 (37.48)	2181 (39.51)	1033 (46.85)
**Physical activity**				
No (0)	724 (6.56)	176 (5.32)	422 (7.64)	126 (5.71)
Moderate level (1–10)	7424 (67.30)	2342 (70.84)	4066 (73.66)	1016 (46.08)
High (11–24)	2883 (26.14)	788 (23.84)	1032 (18.70)	1063 (48.21)
**EBS-SF score, Mean (SD)**	16.45 (4.65)	14.87 (4.63)	16.30 (4.36)	19.19 (4.16)
**Eating Behavior**				
Healthy (7–14)	3712 (33.65)	1579 (47.76)	1856 (33.62)	277 (12.56)
General (15–20)	4865 (44.10)	1297 (39.23)	2560 (46.38)	1008 (45.71)
Unhealthy (21–28)	2454 (22.25)	430 (13.01)	1104 (20.00)	920 (41.73)
**BMI, Mean (SD)**	21.87 (3.18)	21.96 (3.18)	21.91 (3.14)	21.72 (3.30)
**Frequency of computer use**				
Never	2139 (19.39)	705 (21.32)	1157 (20.96)	277 (12.56)
Low (1–3 days per week)	3834 (34.76)	1034 (31.29)	1920 (34.78)	880 (39.91)
Mid (4–5 days per week)	2348 (21.29)	678 (20.51)	1112 (20.14)	558 (25.31)
High (6–7 days per week)	2710 (24.57)	889 (26.88)	1331 (24.12)	490 (22.22)
**Frequency of mobile phone use**				
Never	536 (4.86)	157 (4.75)	285 (5.16)	94 (4.26)
Low (1–3 days per week)	1663 (15.08)	348 (10.53)	730 (13.22)	585 (26.53)
Mid (4–5 days per week)	1432 (12.98)	392 (11.86)	620 (11.23)	420 (19.05)
High (6–7 days per week)	7400 (67.08)	2409 (72.87)	3885 (70.39)	1106 (50.16)

Note: EBS-SF: Short-form Sakata Eating Behavior Scale. Chinese Yuan: CNY.

Table S5. Characteristics of respondents by mental health status and age group

Characteristics	Adolescents and young people (*N* = 3087, 27.98%)			Middle-aged people (*N* = 6797, 61.62%)			Older people (*N* = 1147, 10.40%)		
	No mental health symptom (*N* = 960, 31.10%)	Only one symptom (*N* = 1399, 45.32%)	Two or more symptoms (*N* = 728, 23.58%)	No mental health symptom (*N* = 1909, 28.08%)	Only one symptom (*N* = 3605, 53.04%)	Two or more symptoms (*N* = 1283, 18.88%)	No mental health symptom (*N* = 437, 38.10%)	Only one symptom (*N* = 516, 44.99%)	Two or more symptoms (*N* = 194, 16.91%)
**Sex**									
Male	407 (42.40)	582 (41.60)	314 (43.13)	889 (46.57)	1619 (44.91)	641 (49.96)	230 (52.63)	252 (48.84)	99 (51.03)
Female	553 (57.60)	817 (58.40)	414 (56.87)	1020 (53.43)	1986 (55.09)	642 (50.04)	207 (47.37)	264 (51.16)	95 (48.97)
**Nationality**									
The Han	898 (93.54)	1309 (93.57)	676 (92.86)	1834 (96.07)	3385 (93.90)	1206 (94.00)	426 (97.48)	478 (92.64)	174 (89.69)
Others	62 (6.46)	90 (6.43)	52 (7.14)	75 (3.93)	220 (6.10)	77 (6.00)	11 (2.52)	38 (7.36)	20 (10.31)
**Religious belief**									
No	944 (98.33)	1365 (97.57)	713 (97.94)	1859 (97.38)	3496 (96.98)	1236 (96.34)	419 (95.88)	494 (95.74)	183 (94.33)
Yes	16 (1.67)	34 (2.43)	15 (2.06)	50 (2.62)	109 (3.02)	47 (3.66)	18 (4.12)	22 (4.26)	11 (5.67)
**Educational level**									
Primary school and below	68 (7.08)	41 (2.93)	16 (2.20)	101 (5.29)	266 (7.38)	129 (10.05)	181 (41.42)	243 (47.09)	82 (42.27)
Middle school	253 (26.35)	369 (26.38)	168 (23.08)	567 (29.70)	1259 (34.92)	370 (28.84)	169 (38.67)	195 (37.79)	67 (34.54)
College/university degree or above	639 (66.57)	989 (70.69)	544 (74.72)	1241 (65.01)	2080 (57.70)	784 (61.11)	87 (19.91)	78 (15.12)	45 (23.19)
**Residential areas**									
Urban	670 (69.79)	997 (71.27)	491 (67.45)	1491 (78.10)	2730 (75.73)	975 (75.99)	258 (59.04)	285 (55.23)	111 (57.22)
Rural	290 (30.21)	402 (28.73)	237 (32.55)	418 (21.90)	875 (24.27)	308 (24.01)	179 (40.96)	231 (44.77)	83 (42.78)
**The number of residential properties owned by family**									
No	88 (9.17)	136 (9.72)	128 (17.58)	118 (6.18)	323 (8.96)	200 (15.59)	29 (6.64)	35 (6.78)	26 (13.40)
Only one	524 (54.58)	787 (56.25)	383 (52.61)	1137 (59.56)	2235 (62.00)	773 (60.25)	278 (63.62)	363 (70.35)	118 (60.82)
More than or equal to two	348 (36.25)	476 (34.03)	217 (29.81)	654 (34.26)	1047 (29.04)	310 (24.16)	130 (29.74)	118 (22.87)	50 (25.78)
**Household indebtedness**									
No	577 (60.10)	793 (56.68)	415 (57.01)	1168 (61.18)	2190 (60.75)	696 (54.25)	380 (86.96)	420 (81.40)	141 (72.68)
Yes	383 (39.90)	606 (43.32)	313 (42.99)	741 (38.82)	1415 (39.25)	587 (45.75)	57 (13.04)	96 (18.60)	53 (27.32)
**Monthly household income per capita (CNY)**									
Low (≤ 3000)	320 (33.33)	468 (33.45)	287 (39.42)	386 (20.22)	957 (26.55)	357 (27.83)	154 (35.24)	233 (45.16)	84 (43.30)
General (3001–6000)	337 (35.10)	526 (37.60)	240 (32.97)	745 (39.03)	1519 (42.14)	481 (37.49)	167 (38.22)	180 (34.88)	59 (30.41)
Middle (6001–8999)	155 (16.15)	204 (14.58)	93 (12.77)	406 (21.27)	584 (16.20)	241 (18.78)	80 (18.31)	67 (12.98)	30 (15.46)

High (≥ 9000)	148 (15.42)	201 (14.37)	108 (14.84)	372 (19.48)	545 (15.11)	204 (15.90)	36 (8.23)	36 (6.98)	21 (10.83)
**Number of siblings**									
No	351 (36.56)	514 (36.74)	255 (35.03)	358 (18.75)	688 (19.08)	314 (24.47)	29 (6.64)	39 (7.56)	16 (8.25)
One or more	609 (63.44)	885 (63.26)	473 (64.97)	1551 (81.25)	2917 (80.92)	969 (75.53)	408 (93.36)	477 (92.44)	178 (91.75)
**Smoking status**									
Non-smokers or ex-smokers	925 (96.35)	1338 (95.64)	678 (93.13)	1611 (84.39)	3061 (84.91)	1037 (80.83)	381 (87.19)	442 (85.66)	159 (81.96)
Current smokers	35 (3.65)	61 (4.36)	50 (6.87)	298 (15.61)	544 (15.09)	246 (19.17)	56 (12.81)	74 (14.34)	35 (18.04)
**Alcohol consumption in the past year**									
No	640 (66.67)	872 (62.33)	391 (53.71)	1130 (59.19)	2111 (58.56)	653 (50.9)	297 (67.96)	356 (68.99)	128 (65.98)
Yes	320 (33.33)	527 (37.67)	337 (46.29)	779 (40.81)	1494 (41.44)	630 (49.1)	140 (32.04)	160 (31.01)	66 (34.02)
**Physical activity**									
No (0)	51 (5.31)	78 (5.58)	35 (4.81)	89 (4.66)	285 (7.91)	65 (5.07)	36 (8.24)	59 (11.43)	26 (13.40)
Moderate level (1–10)	631 (65.73)	998 (71.34)	337 (46.29)	1343 (70.35)	2649 (73.48)	582 (45.36)	368 (84.21)	419 (81.20)	97 (50.00)
High (11–24)	278 (28.96)	323 (23.09)	356 (48.9)	477 (24.99)	671 (18.61)	636 (49.57)	33 (7.55)	38 (7.37)	71 (36.60)
**EBS-SF score, Mean (SD)**	15.99 (4.42)	17.19 (4.31)	19.70 (4.21)	14.80 (4.61)	16.22 (4.29)	19.16 (4.01)	12.70 (4.42)	14.50 (4.38)	17.52 (4.49)
**Eating Behavior**									
Healthy (7–14)	346 (36.04)	369 (26.38)	78 (10.71)	930 (48.72)	1209 (33.54)	153 (11.93)	303 (69.34)	278 (53.88)	46 (23.71)
General (15–20)	453 (47.19)	675 (48.25)	315 (43.27)	740 (38.76)	1706 (47.32)	599 (46.69)	104 (23.80)	179 (34.69)	94 (48.45)
Unhealthy (21–28)	161 (16.77)	355 (25.37)	335 (46.02)	239 (12.52)	690 (19.14)	531 (41.38)	30 (6.86)	59 (11.43)	54 (27.84)
**BMI, Mean (SD)**	20.66 (3.05)	20.72 (3.12)	20.81 (3.12)	22.52 (3.08)	22.36 (3.02)	22.21 (3.27)	22.37 (3.03)	22.00 (3.18)	21.87 (3.42)
**Frequency of computer use**									
Never	103 (10.73)	106 (7.58)	56 (7.69)	301 (15.77)	727 (20.17)	143 (11.15)	301 (68.88)	324 (62.79)	78 (40.21)
Low (1–3 days per week)	312 (32.50)	497 (35.53)	280 (38.46)	629 (32.95)	1297 (35.98)	531 (41.39)	93 (21.28)	126 (24.42)	69 (35.57)
Mid (4–5 days per week)	224 (23.33)	352 (25.16)	183 (25.14)	432 (22.63)	720 (19.97)	340 (26.50)	22 (5.03)	40 (7.75)	35 (18.04)
High (6–7 days per week)	321 (33.44)	444 (31.73)	209 (28.71)	547 (28.65)	861 (23.88)	269 (20.96)	21 (4.81)	26 (5.04)	12 (6.18)
**Frequency of mobile phone use**									
Never	22 (2.29)	28 (2.00)	18 (2.47)	25 (1.31)	89 (2.47)	32 (2.49)	110 (25.17)	168 (32.56)	44 (22.68)
Low (1–3 days per week)	109 (11.35)	196 (14.01)	166 (22.80)	150 (7.86)	403 (11.18)	343 (26.73)	89 (20.37)	131 (25.39)	76 (39.18)
Mid (4–5 days per week)	81 (8.44)	134 (9.58)	126 (17.31)	238 (12.47)	417 (11.57)	256 (19.95)	73 (16.70)	69 (13.37)	38 (19.59)
High (6–7 days per week)	748 (77.92)	1041 (74.41)	418 (57.42)	1496 (78.36)	2696 (74.78)	652 (50.83)	165 (37.76)	148 (28.68)	36 (18.56)

Note: EBS-SF: Short-form Sakata Eating Behavior Scale. Chinese Yuan: CNY.

Table S6. Accuracy of random forest models in different age and sex groups

Groups	Accuracy	
	One mental health symptom vs. No	Comorbid vs. No
**Adolescents and young people**		
Overall	0.60	0.70
Male	0.62	0.74
Female	0.59	0.69
**Middle-aged people**		
Overall	0.66	0.73
Male	0.66	0.75
Female	0.66	0.75
**Older people**		
Overall	0.59	0.82
Male	0.56	0.82
Female	0.59	0.76

Table S7. The association between electronic device use and comorbid mental health symptoms in different age groups

	No mental health symptom	Only one symptom		Two or more symptoms	
	n **(%)**	***n*** **(%)**	**OR (95% CI)**	***n*** **(%)**	**OR (95% CI)**
**Age group 1: Adolescents and young people (*N* = 3087, 27.98%)**	***n*** **= 960, 31.10%**	***n*** **= 1399, 45.32%**		***n*** **= 728, 23.58%**	
**Frequency of computer use**					
Never	103 (10.73)	106 (7.58)	ref	56 (7.69)	ref
Low (1–3 days per week)	312 (32.5)	497 (35.53)	1.32 (0.95-1.83)	280 (38.46)	1.05 (0.70-1.58)
Mid (4–5 days per week)	224 (23.33)	352 (25.16)	1.32 (0.93-1.87)	183 (25.14)	0.96 (0.62-1.48)
High (6–7 days per week)	321 (33.44)	444 (31.74)	1.18 (0.84-1.67)	209 (28.71)	0.82 (0.54-1.26)
**Frequency of mobile phone use**					
Never	22 (2.91)	28 (2.00)	ref	18 (2.47)	ref
Low (1–3 days per week)	109 (11.35)	196 (14.01)	1.04 (0.55-1.99)	166 (22.80)	0.83 (0.39-1.78)
Mid (4–5 days per week)	81 (8.44)	134 (9.58)	0.97 (0.50-1.89)	126 (17.31)	1.01 (0.46-2.19)
High (6–7 days per week)	748 (77.92)	1041 (74.41)	0.72 (0.39-1.34)	418 (57.42)	**0.38 (0.18-0.78)****
**Age group 2: Middle-aged people (*N* = 6797, 61.62%)**	***n*** **= 1909, 28.09%**	***n*** **= 3605, 53.04%**		***n*** **= 1283, 18.87%**	
**Frequency of computer use**					
Never	301 (15.77)	727 (20.17)	ref	143 (11.15)	ref
Low (1–3 days per week)	629 (32.95)	1297 (35.98)	0.9,998 (0.83-1.20)	531 (41.39)	**1.63 (1.25-2.11)*****
Mid (4–5 days per week)	432 (22.63)	720 (19.97)	0.85 (0.69-1.04)	340 (26.50)	**1.48 (1.11-1.97)****
High (6–7 days per week)	547 (28.65)	861 (23.88)	0.83 (0.68-1.01)	269 (20.96)	1.005 (0.75-1.35)
**Frequency of mobile phone use**					
Never	25 (1.31)	89 (2.47)	ref	32 (2.49)	ref
Low (1–3 days per week)	150 (7.86)	403 (11.18)	0.81 (0.49-1.33)	343 (26.73)	1.23 (0.67-2.24)
Mid (4–5 days per week)	238 (12.47)	417 (11.57)	**0.56 (0.35-0.91)***	256 (19.95)	0.67 (0.37-1.21)
High (6–7 days per week)	1496 (78.37)	2696 (74.79)	**0.58 (0.37-0.92)***	652 (50.83)	**0.36 (0.20-0.65)****
**Age group 3: Older people (*N* = 1147, 10.40%)**	***n*** **= 437, 38.10%**	***n*** **= 516, 44.99%**		***n*** **= 194, 16.91%**	
**Frequency of computer use**					
Never	301 (68.88)	324 (62.79)	ref	78 (40.21)	ref
Low (1–3 days per week)	93 (21.28)	126 (24.42)	**1.72 (1.18-2.51)****	69 (35.57)	**2.69 (1.58-4.60)*****
Mid (4–5 days per week)	22 (5.03)	40 (7.75)	**2.11 (1.14-3.91)***	35 (18.04)	**3.43 (1.62-7.29)****
High (6–7 days per week)	21 (4.81)	26 (5.04)	1.87 (0.95-3.68)	12 (6.19)	1.67 (0.64-4.40)
**Frequency of mobile phone use**					
Never	110 (25.17)	168 (32.56)	ref	44 (22.68)	ref
Low (1–3 days per week)	89 (20.37)	131 (25.39)	0.91 (0.61-1.36)	76 (39.18)	1.15 (0.65-2.02)
Mid (4–5 days per week)	73(16.70)	69 (13.37)	**0.63 (0.40-0.99)***	38 (19.59)	0.79 (0.41-1.51)
High (6–7 days per week)	165 (37.76)	148 (28.68)	**0.61 (0.41-0.90)***	36 (18.56)	**0.37 (0.20-0.69)****

Note: Reference group: No mental health symptom. Adjusted by gender, nationality, religious belief, educational level, residential areas, the number of residential properties owned by family, household indebtedness, monthly household income per capita, number of siblings, smoking status, alcohol consumption, BMI, physical activity, and eating behavior. * $p < 0.05$, ** $p < 0.01$, *** $p < 0.001$.

Table S8. The association between electronic device use and comorbid mental health symptoms in different age and sex groups

	Male					Female				
	No mental health symptom	Only one symptom		Two or more symptoms		No mental health symptom	Only one symptom		Two or more symptoms	
	n (%)	*n* (%)	OR (95% CI)	*n* (%)	OR (95% CI)	*n* (%)	*n* (%)	OR (95% CI)	*n* (%)	OR (95% CI)
**Age group 1: Adolescents and young people (*N* = 3087, 27.98%)**	***n* = 1303, 42.21%**					***n* = 1784, 57.79%**				
	***n* = 407, 31.24%**	***n* = 582, 44.67%**		***n* = 314, 24.09%**		***n* = 553, 31.00%**	***n* = 817, 45.80%**		***n* = 414, 23.20%**	
**Frequency of computer use**										
Never	57 (14.00)	50 (8.59)	ref	27 (8.60)	ref	46 (8.32)	56 (6.85)	ref	29 (7.00)	ref
Low (1–3 days per week)	115 (28.26)	203 (34.88)	**1.76 (1.08-2.84)***	118 (37.58)	1.09 (0.60-1.99)	197 (35.62)	294 (35.99)	0.97 (0.61-1.55)	162 (39.13)	0.97 (0.55-1.71)
Mid (4–5 days per week)	81 (19.90)	111 (19.07)	1.42 (0.84-2.40)	74 (23.57)	0.99 (0.52-1.90)	143 (25.86)	241 (29.50)	1.12 (0.69-1.82)	109 (26.33)	0.91 (0.50-1.66)
High (6–7 days per week)	154 (37.84)	218 (37.46)	1.42 (0.87-2.33)	95 (30.25)	0.69 (0.37-1.29)	167 (30.20)	226 (27.66)	0.92 (0.56-1.50)	114 (27.54)	0.89 (0.49-1.62)
**Frequency of mobile phone use**										
Never	10 (2.46)	15 (2.58)	ref	11 (3.50)	ref	12 (2.17)	13 (1.59)	ref	7 (1.69)	ref
Low (1–3 days per week)	52 (12.78)	114 (19.59)	1.10 (0.43-2.82)	90 (28.66)	0.57 (0.18-1.75)	57 (10.31)	82 (10.04)	1.03 (0.41-2.56)	76 (18.36)	1.15 (0.38-3.45)
Mid (4–5 days per week)	41 (10.07)	55 (9.45)	0.75 (0.29-1.99)	62 (19.75)	0.66 (0.21-2.08)	40 (7.23)	79 (9.67)	1.33 (0.52-3.39)	64 (15.46)	1.52 (0.50-4.63)
High (6–7 days per week)	304 (74.69)	398 (68.38)	0.63 (0.26-1.54)	151 (48.09)	**0.22 (0.07-0.64)****	444 (80.29)	643 (78.70)	0.91 (0.38-2.16)	267 (64.49)	0.63 (0.22-1.81)
**Age group 2: Middle-aged people (*N* = 6797, 61.62%)**	***n* = 3149, 46.33%**					***n* = 3648, 53.67%**				
	***n* = 889, 28.23%**	***n* = 1619, 51.41%**		***n* = 641, 20.36%**		***n* = 1020, 27.96%**	***n* = 1986, 54.44%**		***n* = 642, 17.60%**	
**Frequency of computer use**										
Never	133 (14.96)	324 (20.01)	ref	73 (11.39)	ref	168 (16.47)	403 (20.29)	ref	70 (10.90)	ref
Low (1–3 days per week)	289 (32.51)	583 (36.01)	1.04 (0.80-1.37)	277 (35.41)	**1.91 (1.30-2.79)****	340 (33.33)	714 (25.95)	0.97 (0.76-1.24)	254 (39.56)	1.43 (0.989-2.06)
Mid (4–5 days per week)	200 (22.50)	326 (20.14)	0.91 (0.67-1.23)	162 (25.27)	**1.55 (1.02-2.36)***	232 (22.75)	394 (19.84)	0.83 (0.63-1.09)	178 (27.73)	1.44 (0.96-2.16)
High (6–7 days per week)	267 (3,003)	386 (23.84)	0.81 (0.60-1.09)	129 (20.13)	1.03 (0.67-1.57)	280 (27.45)	475 (23.92)	0.86 (0.66-1.14)	140 (21.81)	1.01 (0.67-1.53)
**Frequency of mobile phone use**										
Never	13 (1.46)	54 (3.34)	ref	24 (3.74)	ref	12 (1.18)	35 (1.76)	ref	8 (1.25)	ref
Low (1–3 days per week)	76 (8.55)	200 (12.35)	0.69 (0.35-1.38)	189 (29.49)	1.01 (0.46-2.23)	74 (7.25)	203 (10.22)	0.97 (0.47-1.99)	154 (23.99)	1.98 (0.72-5.47)
Mid (4–5 days per week)	124 (13.95)	201 (12.42)	**0.46 (0.23-0.90)***	147 (22.93)	0.57 (0.26-1.24)	114 (11.18)	216 (10.88)	0.72 (0.35-1.47)	109 (16.98)	1.06 (0.39-2.93)

High (6–7 days per week)	676 (76.04)	1164 (71.89)	**0.49 (0.26-0.93)***	281 (43.84)	**0.26 (0.12-0.55)*****	820 (80.39)	1532 (77.14)	0.72 (0.36-1.42)	371 (57.78)	0.69 (0.26-1.85)
**<u>Age group 3</u>: Older people**	***n* = 581, 50.65%**					***n* = 566, 49.35%**				
**(*N* = 1147, 10.40%)**	***n* = 230, 39.59%**	***n* = 252, 43.37%**		***n* = 99, 17.04%**		***n* = 207, 36.57%**	***n* = 264, 46.64%**		***n* = 95, 16.78%**	
**Frequency of computer use**										
Never	151 (65.65)	148 (58.73)	ref	32 (32.32)	ref	150 (72.46)	176 (66.67)	ref	46 (48.42)	ref
Low (1–3 days per week)	57 (24.78)	73 (28.97)	**1.77 (1.07-2.94)***	37 (37.37)	**4.25 (2.02-8.96)*****	36 (17.39)	53 (20.08)	1.72 (0.95-3.10)	32 (33.68)	1.85 (0.81-4.21)
Mid (4–5 days per week)	8 (3.48)	19 (7.53)	**2.77 (1.07-7.17)***	23 (23.23)	**9.79 (3.21-29.89)*****	14 (6.769)	21 (7.95)	1.68 (0.71-4.00)	12 (12.63)	1.03 (0.32-3.29)
High (6–7 days per week)	14 (6.09)	12 (4.76)	1.48 (0.57-3.82)	7 (7.08)	2.86 (0.76-10.76)	7 (3.38)	14 (5.30)	2.43 (0.85-6.95)	5 (5.26)	1.42 (0.30-6.65)
**Frequency of mobile phone use**										
Never	54 (23.48)	64 (25.40)	ref	18 (18.18)	ref	56 (27.05)	104 (39.39)	ref	26 (27.37)	ref
Low (1–3 days per week)	47 (20.43)	69 (27.38)	1.18 (0.67-2.10)	44 (44.44)	1.92 (0.85-4.35)	42 (20.29)	62 (23.48)	0.66 (0.37-1.17)	32 (33.68)	0.66 (0.29-1.53)
Mid (4–5 days per week)	44 (19.13)	34 (13.49)	0.66 (0.35-1.24)	22 (22.22)	0.95 (0.37-2.40)	29 (14.01)	35 (13.26)	0.59 (0.30-1.16)	16 (16.84)	0.63 (0.24-1.67)
High (6–7 days per week)	85 (36.96)	85 (33.73)	0.91 (0.53-1.56)	15 (15.16)	**0.36 (0.14-0.91)***	80 (38.65)	63 (23.87)	**0.36 (0.20-0.65)****	21 (22.11)	**0.36 (0.15-0.87)***

Note: Reference group: No mental health symptom. Adjusted by nationality, religious belief, educational level, residential areas, the number of residential properties owned by family, household indebtedness, monthly household income per capita, number of siblings, smoking status, alcohol consumption, BMI, physical activity, and eating behavior. * $p < 0.05$, ** $p < 0.01$, *** $p < 0.001$.
